# Antiviral RISC mainly targets viral mRNA but not genomic RNA of tospovirus

**DOI:** 10.1371/journal.ppat.1009757

**Published:** 2021-07-28

**Authors:** Hao Hong, Chunli Wang, Ying Huang, Min Xu, Jiaoling Yan, Mingfeng Feng, Jia Li, Yajie Shi, Min Zhu, Danyu Shen, Peijun Wu, Richard Kormelink, Xiaorong Tao

**Affiliations:** 1 Key Laboratory of Plant Immunity, Department of Plant Pathology, Nanjing Agricultural University, Nanjing, P. R. China; 2 Financial Department, Nanjing Agricultural University, Nanjing, P. R. China; 3 Laboratory of Virology, Department of Plant Sciences, Wageningen University, Wageningen, The Netherlands; University of Cambridge, UNITED STATES

## Abstract

Antiviral RNA silencing/interference (RNAi) of negative-strand (-) RNA plant viruses (NSVs) has been studied less than for single-stranded, positive-sense (+)RNA plant viruses. From the latter, genomic and subgenomic mRNA molecules are targeted by RNAi. However, genomic RNA strands from plant NSVs are generally wrapped tightly within viral nucleocapsid (N) protein to form ribonucleoproteins (RNPs), the core unit for viral replication, transcription and movement. In this study, the targeting of the NSV tospoviral genomic RNA and mRNA molecules by antiviral RNA-induced silencing complexes (RISC) was investigated, *in vitro* and *in planta*. RISC fractions isolated from tospovirus-infected *N*. *benthamiana* plants specifically cleaved naked, purified tospoviral genomic RNAs *in vitro*, but not genomic RNAs complexed with viral N protein. *In planta* RISC complexes, activated by a tobacco rattle virus (TRV) carrying tospovirus *NSs* or *Gn* gene fragments, mainly targeted the corresponding viral mRNAs and hardly genomic (viral and viral-complementary strands) RNA assembled into RNPs. In contrast, for the (+)ssRNA cucumber mosaic virus (CMV), RISC complexes, activated by TRV carrying CMV *2a* or *2b* gene fragments, targeted CMV genomic RNA. Altogether, the results indicated that antiviral RNAi primarily targets tospoviral mRNAs whilst their genomic RNA is well protected in RNPs against RISC-mediated cleavage. Considering the important role of RNPs in the replication cycle of all NSVs, the findings made in this study are likely applicable to all viruses belonging to this group.

## Author summary

Plants have evolved RNA silencing as the first line of defense against virus infection. Antiviral RNA silencing as well as viral counter-defense strategies have been extensively studied for ss(+)RNA plant viruses, but less for plant negative-strand (-)/ambisense RNA plant viruses (NSVs). Upon virus infection, RNA-induced silencing complexes (RISCs) are activated to bind and cleave invasive viral RNAs. Unlike ss(+)RNA plant viruses, genomic RNA molecules of plant NSVs are not naked and tightly associate with viral nucleocapsid protein into ribonucleoproteins (RNPs), the core infectious unit for NSVs. However, their viral mRNAs are not assembled into RNPs. Whether the assembly of genomic RNA into RNPs provides another counter-defense strategy to protect against antiviral RISC activity has not been determined for any NSV. In this study, using tospovirus as a representative plant NSV model system, it is demonstrated that antiviral RISC mainly targets viral mRNAs but not genomic RNA embedded in RNPs. Our findings suggest that the RNA targets of RISC differ between plant NSVs and ss(+)RNA plant viruses and that RNPs provide another counter-defense way for NSVs to (partially) evade/escape from cytoplasmic RISC-mediated degradation.

## Introduction

Plants have evolved different layers of defense against pathogen invasions [1–3]. One of the first layers involves cell-surface immune receptors that recognize pathogen associated molecular patterns (PAMPs) and induce pattern triggered immunity (PTI). Pathogens, on the other hand, have evolved effector proteins to attack and inhibit PTI to facilitate pathogen infection. In response, plants also evolved intracellular immune receptors that recognize pathogen effectors and induce effector triggered immunity (ETI). This ongoing battle is commonly presented in a zigzag model [1]. RNA silencing (or also referred to as RNA interference (RNAi)) is a highly conserved mechanism in eukaryotes that is also involved in host innate immunity against virus infections [4–9]. In plants, but not in mammals or insects, RNAi is amplified by the action of host encoded RNA-dependent RNA polymerases (RDRs) and enables the mounting of a strong antiviral RNAi response. RNA silencing can be considered a PTI and acts specific to the virus causing the infection. During viral replication, plant viruses generate double-stranded RNA (dsRNA) that result from either replication intermediates or RNA folding structures within viral (m)RNA molecules. Viral dsRNA is a typical PAMP and is the trigger for RNA silencing [10]. DsRNA is recognized and processed by Dicer-like (DCL) proteins of the host plant into small interfering RNA (siRNA) duplexes of 21–24 nt in size [8,9]. These are loaded into an RNA-induced silencing complex (RISCs), containing a core Argonaute effector [11]. After unwinding of the siRNA duplex molecule one strand, the so-called guide strand, uploads into a pre-RISC complex and turns it into an activated RISC. When loaded with viral siRNAs (vsiRNAs), RISC becomes antiviral and is able to surveil for single stranded viral RNA target molecules with sequence complementarity to the guide strand, followed by Ago-mediated cleavage of the target RNA. The isolation of antiviral RISC from virus-infected plants has been reported for *Tobacco rattle virus* (TRV) and *Tomato bushy stunt virus* (TBSV) [12–14], and for TRV shown to specifically cleave TRV RNA *in vitro*. Exogenous addition of RNA containing siRNAs from plants infected with an unrelated virus would re-program the specificity of RISC to target the heterologous virus genome [13]. Interestingly, fractions of plants infected with TBSV P19 mutants, but not from those infected with the wild-type virus, contained the RNAi-associated ssRNA-specific ribonucleases [12].

**Fig 1:**
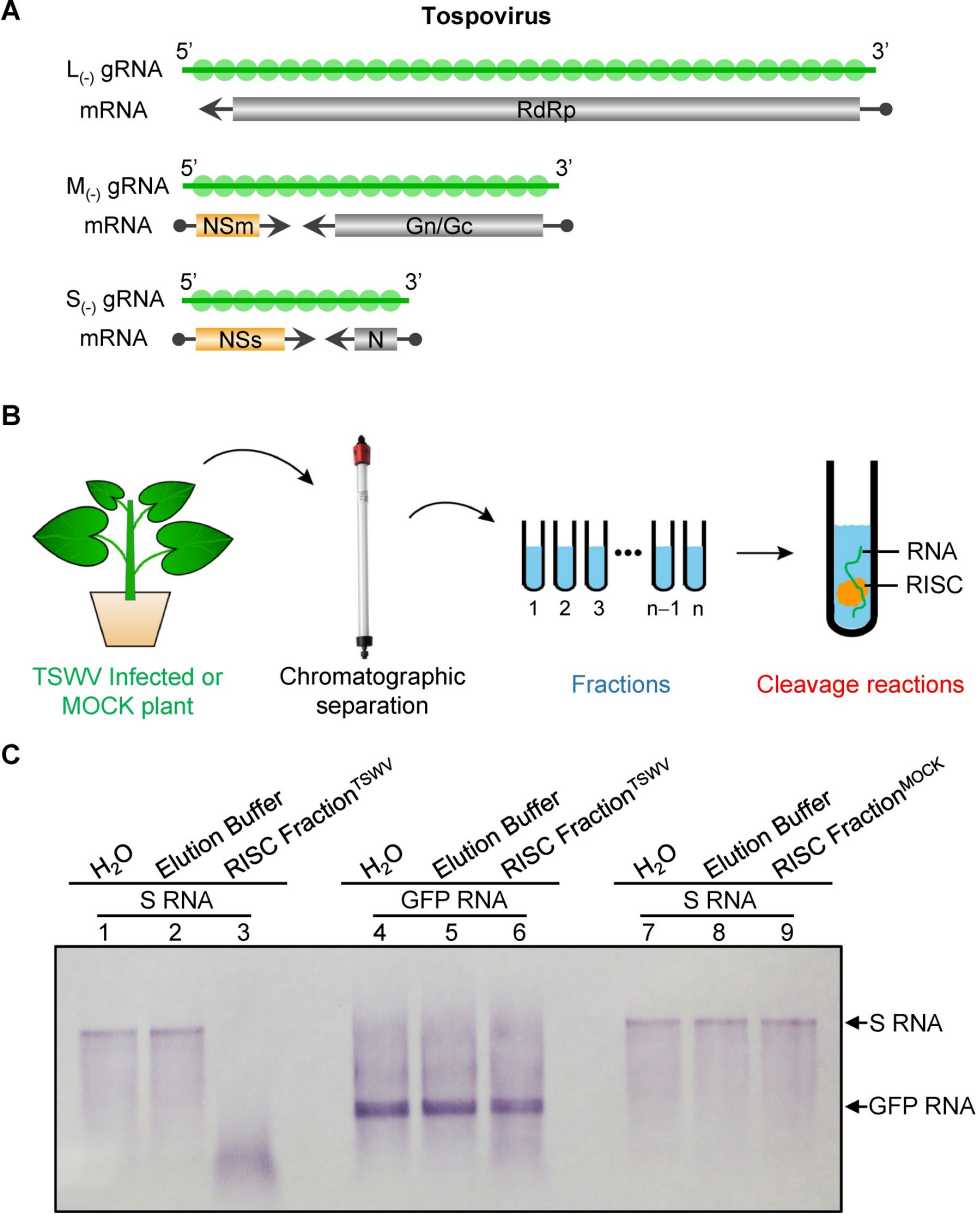
Chromatography fractions isolated from TSWV-infected plant contain RISC associated nuclease activity that specifically cleave S RNA *in vitro*. (A) Schematical diagram of the tospoviral tripartite RNA genome, encoding five open reading frames. Genomic RNA (gRNA) molecules are tightly associated with N protein (in green). Viral transcripts of the five ORFs are aligned below the gRNA strands. Those for the RdRP, Gn/Gc and N (in grey) result from primary transcription of the gRNA, whereas the additional NSm and NSs transcripts from the ambisense M- and S RNA (in orange), respectively, are produced by secondary transcription, i.e. after gRNA has been copied into a complementary strand that serves as a template for secondary transcription (and replication of progeny gRNA). Small dots at the 5’ end of the viral mRNAs present heterogenous, host derived, capped-RNA leader sequence resulting from cap-snatching. Minus sign (+) represents the vc strand of genomic RNA. Minus sign (−) and 3′ to 5′ designation represent the v strand of genomic RNA. (B) Diagram of antiviral RISC isolation and *in vitro* cleavage assay for RISC associated nuclease activity. Leaves were harvested from TSWV-infected or mock-treated *N*. *benthamiana* plant (~40 g). The extract of leaves was loaded onto Hydroxyapatite column followed by Superdex S-200 HR gel column. Sixteen Superdex S-200 fractions were finally collected and every fraction (600 μL) was incubated with *in vitro* transcribed DIG-labelled RNA for the activity of RISC associated nuclease. (C) DIG-labelled full length S genomic RNA was incubated with isolated chromatography fraction 6 (S1A Fig) from TSWV-infected *N*. *benthamiana* plants (Lane 1–3) or with chromatography fraction 6 (S1B Fig) from mock-inoculated control *N*. *benthamiana* plants (Lane 4–6). As a negative control, DIG-labelled full length GFP RNA was incubated with isolated chromatography fraction 6 from TSWV-infected *N*. *benthamiana* plants (Lane 7–9).

Negative-stranded RNA viruses (NSVs) include not only dangerous medical important pathogens but also agronomical important plant pathogens. Some well-known NSVs include Ebola virus (EBOV), rabies virus, influenza A virus (FLUAV) and Rift Valley fever virus (RVFV) [15–17], and cause important diseases in humans. Some plant NSVs such as tospoviruses, tenuiviruses and rhabdoviruses, cause severe problems in agronomic crops. Tospoviruses are amongst the most important plant NSVs in the world, infecting more than 1000 plant species from over 80 families [18], and pose major threats to global food production [19,20]. Tospoviruses are classified in the genus *Orthotospovirus*, family *Tospoviridae* within the order *Bunyavidales* [21], and contain more than thirty well characterized species. Based on sequence identity of the nucleocapsid (N) protein and geographic distribution, species of tospoviruses are grouped into an American- and Euro-Asia clade [20,22]. *Tomato spotted wilt virus* (TSWV) is the type species of *Orthotospovirus* and categorized into the American clade [19,20]. Tospoviruses have a tripartite RNA genome that, according to their size, are named large (L), medium (M) and small (S) RNA (Fig 1A). The L RNA segment is of negative polarity and encodes an RNA-dependent RNA polymerase (RdRp) [23]. The M and S RNAs are ambisense, *i*.*e*. they encode two non-overlapping open reading frames (ORFs) on opposite strands. The M RNA encodes a precursor for the glycoproteins in the viral complementary (vc) strand, which is further processed into the mature Gn and Gc glycoproteins. These are required for virus particle maturation and presented as spikes on the surface of the particle envelope [24–26]. In the viral (v) strand, the M RNA encodes a nonstructural protein (NSm), which is involved in cell-to-cell and long distance movement [27–30]. The S RNA similarly codes for a second non-structural protein (NSs) in the v strand, and the N protein in the vc strand [31]. The NSs is an RNA silencing suppressor (RSS) and acts by binding dsRNA in a size-independent manner [32–35]. In binding vsiRNAs, the NSs protein prevents their systemic movement and suppresses systemic RNA silencing as well [36]. The N protein tightly associates with viral genomic RNA and together with a few copies of the RdRp form the (minimal) infectious RNP units to initiate viral replication and transcription [37–39]. Viral mRNAs of TSWV and other bunyaviruses are not assembled into RNPs [40–42]. The crystal structure of the TSWV N protein has been resolved [43,44] and show an interaction of monomeric N units into trimers. Recently, a reverse genetics (RG) system for TSWV has been established, the first one for a segmented plant NSV, which opens up new possibilities to study the role of viral proteins in the life cycle of TSWV [45].

Antiviral RNA silencing has been extensively studied for (+)ssRNA plant viruses [5,6]. However, less is known on antiviral RNA silencing of plant NSVs. Unlike (+)ssRNA plant viruses that directly use genomic RNA as template for replication, the RNA genome of plant NSVs can only engage in replication/transcription when complexed with N and RdRp into RNPs [46,47]. For plant NSVs RNPs also present the units that move cell-to-cell and spread viral infection long distance [23]. For this reason, viral genomic RNA from NSVs may be well protected against the host antiviral RNAi machinery. In this study, using a tospovirus as a plant segmented NSV model system, antiviral RISC was isolated from TSWV-infected plants and tested on the ability to access and cleave genomic RNA present in viral RNPs *in vitro*. Furthermore, RISC targeting of viral mRNA and genomic RNA was also tested *in planta*. Results show that RISCs mainly target tospoviral mRNAs but not genomic RNA embedded within RNPs.

## Results

### TSWV activated RISC specifically cleaves TSWV S RNA but not GFP RNA *in vitro*

Unlike with ss(+)RNA plant viruses, viral genomic RNA of plant NSVs are not naked and always associated with N protein to form ribonucleoprotein (RNP) complexes [19,48]. We therefore hypothesized that the viral RNP structure would protect the viral genomic RNA from cleavage by host antiviral RISC. Since viral mRNAs do not associate with N protein into RNPs, we expected that RISC would likely target the viral mRNA. Here we used tospovirus as plant segmented NSV model system to test this hypothesis. The genomic organization and expression strategy of a tospovirus are illustrated in Fig 1A.

As a first step, RISC was isolated from TSWV-infected *Nicotiana benthamiana* using Hydroxyapatite column chromatography followed with Superdex S-200 column chromatography as described previously [12]. To determine which fractions contain RISC-associated ribonuclease activity, every fraction was incubated with DIG-labeled full-length RNA transcripts of TSWV S (Fig 1B). The results showed that the Superdex S-200 gel filtration fractions 4–9 and fractions 11–16 from TSWV-infected plants exhibit RISC-associated nuclease activity to S RNA (S1A Fig). To rule out that the purified *N*. *benthamiana* RISC complexes contain nonspecific nuclease activity to S RNA, RISC was also isolated from mock-inoculated *N*. *benthamiana* plants and incubated with DIG-labeled full-length S RNA transcripts of TSWV. As shown in S1B Fig, none of the fractions isolated from mock-inoculated plants were able to cleave the S RNA of TSWV. To test the sequence-specific targeting of the (antiviral) RISC-associated nuclease isolated from tospovirus-infected plants, all purified RISC fractions were also incubated *in vitro* with (heterologous) DIG-labelled RNA transcripts of GFP. The results showed that fractions containing RISC-nuclease activity against TSWV S RNA did not cleave GFP RNA (S1C Fig). Upon repetition of the entire experiment including all controls (and analysed on the same gel), the active RISC fractions again specifically cleaved TSWV S RNA but not GFP RNA *in vitro* (Fig 1C).

### Viral RNPs protect genomic RNA against cleavage by antiviral RISC *in vitro*

**Fig 2:**
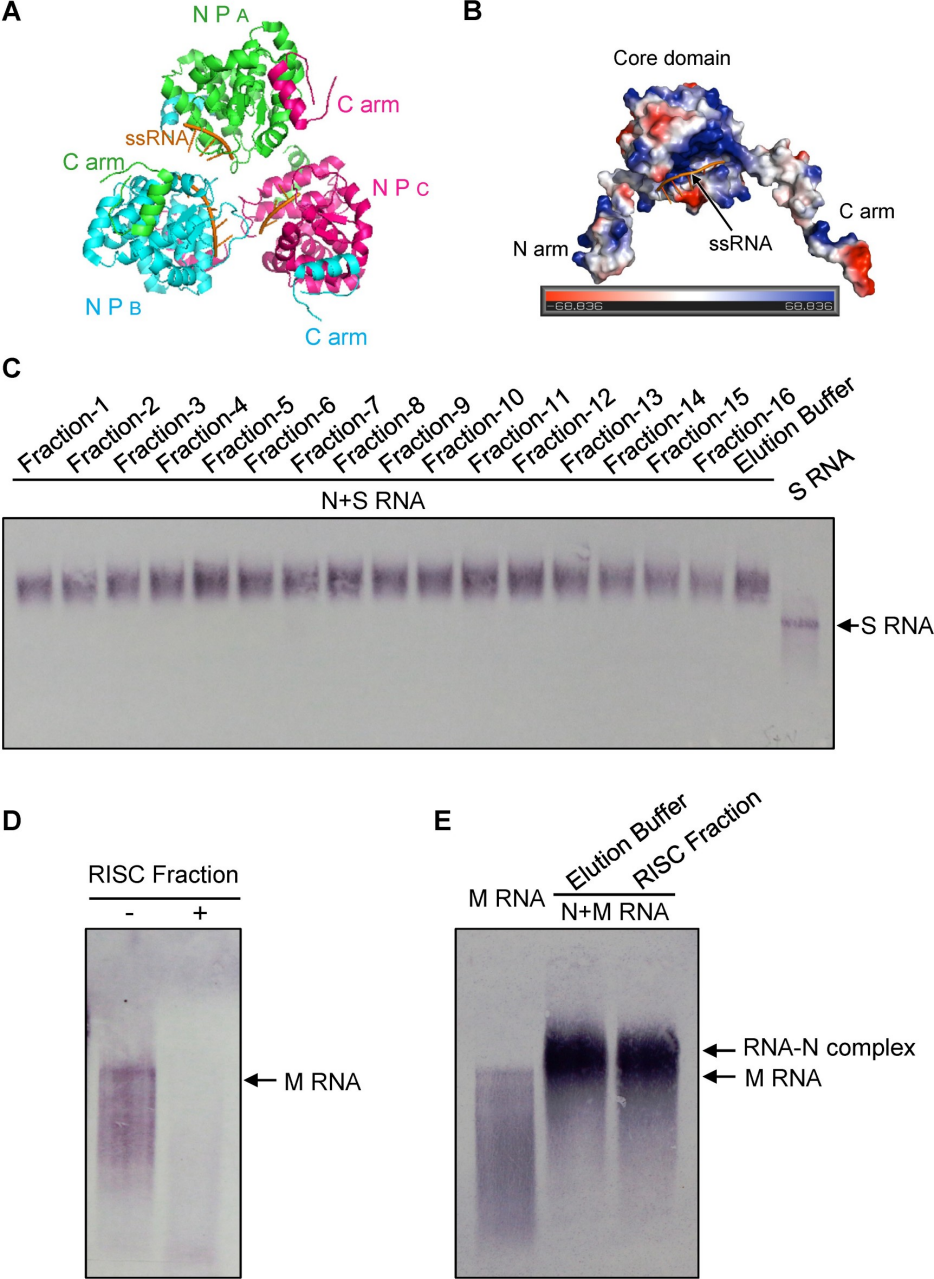
TSWV RNPs protect viral genomic RNA by the cleavage of host antiviral RISC *in vitro*. (A) The carton crystal structure showing trimer of TSWV N:RNA complexes. Protein database bank No. 5ip2 of TSWV N was used to display the structure by PyMol. Chains A, B and C in the trimeric nucleocapsid protein (NP) structure are colored in green, cyan and purple, respectively. (B) The crystal structure of TSWV N-RNA complexes showing the embedding of viral RNA in the cleft of TSWV N protein. (C) *In vitro* cleavage assay of TSWV genomic S RNA complexed with N protein by isolated chromatography fractions from TSWV infected *N*. *benthamiana* plants. DIG-labelled full length genomic S RNA was incubated with TSWV N protein to form N-RNA complexes. All 16 fractions tested in S1A Fig were mixed with N-RNA complexes for the RNA protection assay. (D) *In vitro* cleavage assay of naked genomic M RNA by the fraction containing RISC-activity. DIG-labelled full length M genomic RNA was incubated with fraction 6 (S1A Fig) containing the RISC-activity. (E) *In vitro* cleavage assay of TSWV genomic M RNA complexed with N protein by fraction 6 containing antiviral RISC-activity. DIG-labelled full length M genomic RNA was incubated with TSWV N protein to form N-RNA complexes. The RISC fraction was then added into N-RNA complexes to test the RNA protection activity. The signals on the blot were detected by AP-labeled anti-digoxigenin antibodies and followed with BCIP/NBT staining.

The crystal structure of the TSWV N-RNA complex as earlier resolved, and representative for RNPs [43,44], indicated that the N protein associates into trimers (Fig 2A) and in which the viral genomic RNA is deeply embedded in a cleft of the N protein (Fig 2B). To investigate whether the genomic RNA within RNPs was sufficiently protected against RISC-activity isolated from TSWV-infected plants, DIG-labelled full-length S RNA transcripts were first incubated with TSWV N protein to form N-RNA complexes and next incubated with Superdex S-200 fractions. However, fractions 4–9 and fractions 11–16, which earlier cleaved S RNA *in vitro*, were unable to cleave the viral genomic S RNA complexed with TSWV N protein (Fig 2C). Although the intensity of bands reflecting S RNA:N complexes became slightly weaker after the addition of fractions 14–16, the majority of S RNA:N complexes was still there in the presence of anti-TSWV RISC. These data suggested that the genomic S RNA becomes well protected against degradation by host antiviral RISC *in vitro* after association with N protein. To rule out the possibility that the N protein itself inhibited RISC activity, the experiment was repeated but this time with a previously identified N^R94A/R95A^ mutant compromised in viral RNA binding [39]. When DIG-labeled full-length S RNA transcripts were mixed with high amounts of N^R94A/R95A^ protein, S RNA:N^R94A/R95A^ complexes were formed. However, anti-TSWV RISC fractions were able to degrade the viral S RNA complexed with N^R94A/R95A^ (S2 Fig).

To further characterize the sequence specific targeting of TSWV by antiviral RISC, genomic M RNA of TSWV /or RNA complexed with N protein was subjected to the *in vitro* RISC cleavage assay. When fraction 6, containing RISC activity (and hereafter named “RISC fraction”) was incubated with DIG-labelled full-length M RNA of TSWV, the band of full-length M RNA was completely digested (Fig 2D). When, prior to the incubation with the RISC fraction, DIG-labelled full-length M RNA was incubated with N protein to form N-RNA complexes, the genomic M RNA was resistant to cleavage (Fig 2E). These results further strengthened the first observations, made on the S RNA, that antiviral RISC complexes purified from TSWV-infected *N*. *benthamiana* were able to *in vitro* degrade naked TSWV genomic RNA but not when complexed into viral RNPs.

### TRV-induced RISC activity against TSWV mainly targets viral mRNA but not genomic RNA of TSWV *in planta*

Given that RISC was able to access naked TSWV genomic RNA but not the embedded genomic RNA within RNPs *in vitro*, next TSWV targeting of RISC *in planta* was analyzed. Recently, using an established reverse genetics system for TSWV, the N protein (resulting after primary transcription from the S gRNA, Fig 1A) was shown to be essential for viral replication and transcription, while the NSs protein (expressed after replication and secondary transcription of the S gRNA, Fig 1A) was dispensable [47]. To assess RISC targeting of TSWV RNA *in vivo*, an approach was implemented in which *N*. *benthamiana* were first infected with tobacco rattle virus (TRV), carrying gene fragments of *N* and *NSs*, respectively, to *a priori* assemble antiviral RISC *in planta*, and subsequently challenged with TSWV (S3 Fig). We hypothesized that when RISC activated from TRV-N and TRV-NSs targets the genomic v (or vc) TSWV S RNA molecules, leading to their degradation, it would abrogate viral replication and no TSWV infection would be established in *N*. *benthamiana* plants. In case RISC only targets the viral mRNA, the outcome of the experiment would depend on the viral gene targeted. Considering the role of N in replication, RISC targeting of *N* gene transcripts would inhibit virus replication/infection, while targeting those of *NSs*, being dispensable, would not affect replication and allow the establishment of a viral infection.

**Fig 3:**
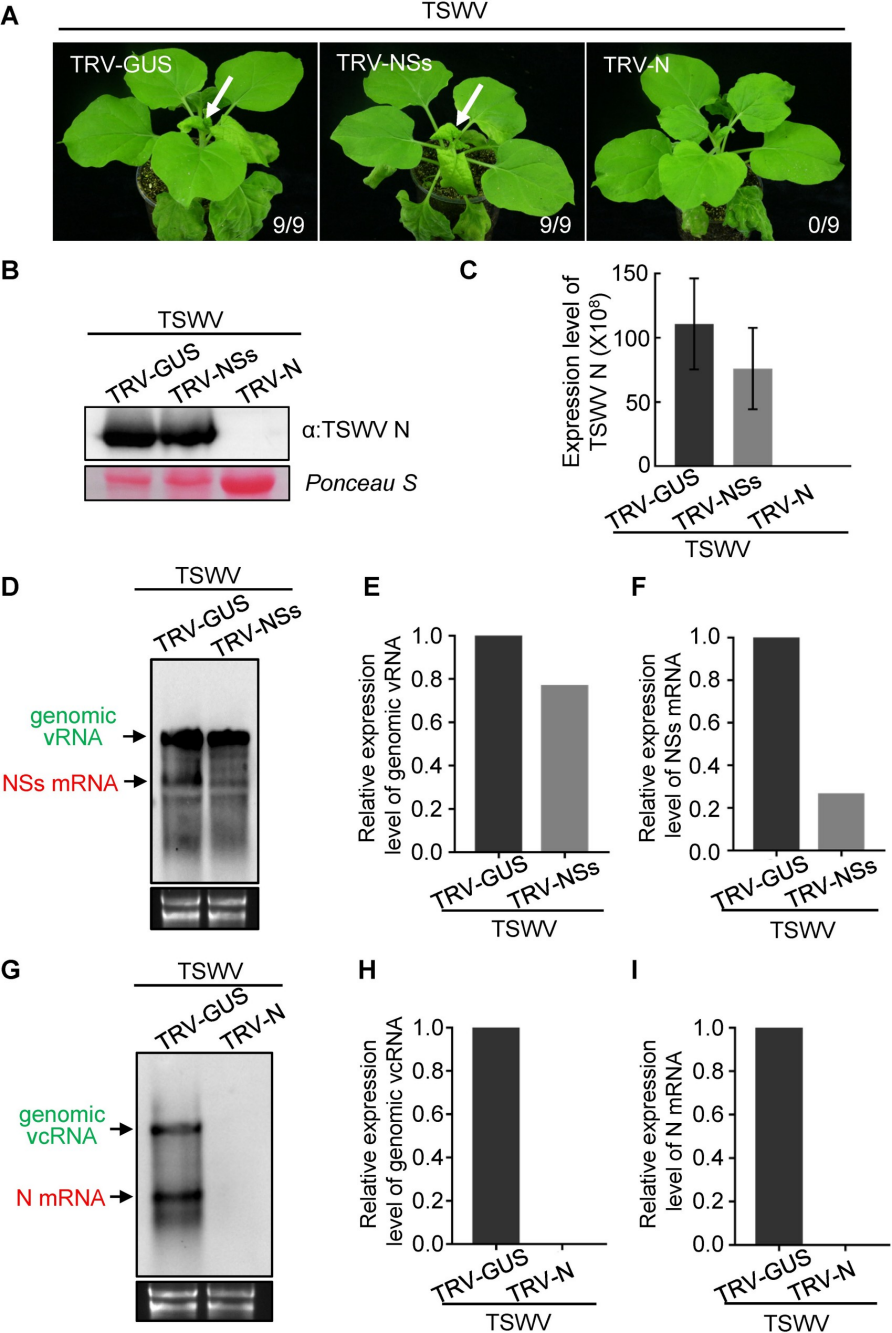
TRV-induced RISC mainly targets viral mRNA but not genomic RNA of TSWV S segment *in planta*. (A) The TRV-NSs, TRV-N and TRV-GUS constructs were used to infect *N*. *benthamiana* plants by agro-infiltration, respectively. At 10 days post agroinfitration, the leaves of TRV-NSs, TRV-N and TRV-GUS pre-infected plants were rub inoculated with sap of TSWV from freshly-infected leaves. The phenotype of TSWV infection was monitored and photographed at 9 days post TSWV inoculation. The white arrow indicates the typical TSWV symptoms in systemically-infected leaves of *N*. *benthamiana* plants. The numbers showing in the image are the total number of the inoculated plants versus the infected plants observed from the treatments. (B) and (C) Detection of TSWV accumulation in systemically-infected leaves of TRV-NSs, TRV-N and TRV-GUS pre-infected *N*. *benthamiana* plant by western blot and qRT-PCR analysis, respectively. TSWV N-specific antibody and TSWV-N specific primers were used to detect accumulation of TSWV N protein and viral RNAs, respectively. The Ponceau S stained gel was used to show the sample loadings. Error bars represent SD (n = 3). (D) Northern blot analysis of genomic vRNA, vcRNA and NSs mRNA of TSWV S segment targeted by pre-assembled RISC from TRV-GUS and TRV-NSs in *N*. *benthamiana* plants using strand specific DIG-labelled NSs probe. Samples were collected at 3 dpi from TSWV inoculated leaves of TRV-GUS and TRV-NSs pre-infected plants. Genomic vRNA and viral NSs mRNA bands are indicated by the arrows in green and red, respectively. Ethidium bromide staining was used to show RNA loading. (E) and (F) Quantification of the accumulation level of genomic vRNA and NSs mRNA, respectively, shown in Fig 3D. (G) Northern blot analysis of genomic vcRNA and N mRNA of TSWV S segment targeted by pre-assembled RISC from TRV-GUS and TRV-N in *N*. *benthamiana* plants using a strand-specific DIG-labeled N probe. Samples were collected at 3 dpi from TSWV inoculated leaves of TRV-GUS and TRV-N pre-infected plants. Genomic vcRNA and N mRNA bands are indicated by the arrows in green and red, respectively. Ethidium bromide staining was used to show RNA loading. (H) and (I) Quantification of the accumulation level of genomic vcRNA and N mRNA, respectively, shown in Fig 3G. All experiments were repeated more than three times.

To test this hypothesis, a 300 bp gene fragment of *N* and *NSs*, respectively, were cloned into TRV. A similar sized fragment of GUS was inserted into TRV and used as a negative control. *N*. *benthamiana* plants were agroinoculated with the TRV constructs and after about 10 days, to allow for assembly of antiviral RISC, the local leaves were challenged with TSWV. Whereas at 9 days post inoculation (dpi) typical TSWV symptoms (Fig 3A, arrow) were observed in systemically-infected leaves from the TRV-NSs pre-treated plants and the TRV-GUS control, no symptoms was discerned in the TRV-N pre-treated plants. Western blot and qRT-PCR analysis confirmed the presence of TSWV in systemically-infected leaves of TRV-GUS and TRV-NSs pre-treated plants but the virus was not detected in the TRV-N pre-treated plants (Fig 3B and 3C). Next, the production levels of genomic S RNA and viral N/NSs mRNA were determined in TRV-GUS, TRV-NSs and TRV-N treated plants. To this end, total RNA was extracted and analyzed by northern blot analysis using NSs/N strand-specific probes to detect NSs mRNA and S vRNA molecules (of similar polarity), or N mRNA and S vcRNA (of similar polarity), respectively. In TRV-NSs pre-infected plants the accumulation of NSs mRNA was significantly lower than in TRV-GUS pre-treated plants (Fig 3D and 3F), whereas the accumulation of genomic S vRNA in TRV-NSs pre-infected plants was only slightly lower than in TRV-GUS pre-infected plants (Fig 3D and 3E). When using the strand specific probe for N, genomic S vcRNA and N mRNA was readily detected in TRV-GUS pre-infected plants (Fig 3G–3I), but not in TRV-N pre-infected plants (Fig 3G–3I). When the experiments were repeated, but the TSWV challenging performed at 15 days after the TRV-GUS, TRV-NSs or TRV-N treatment in *N*. *benthamina* plants, similar results were obtained (S4A and S4B Fig).

**Fig 4:**
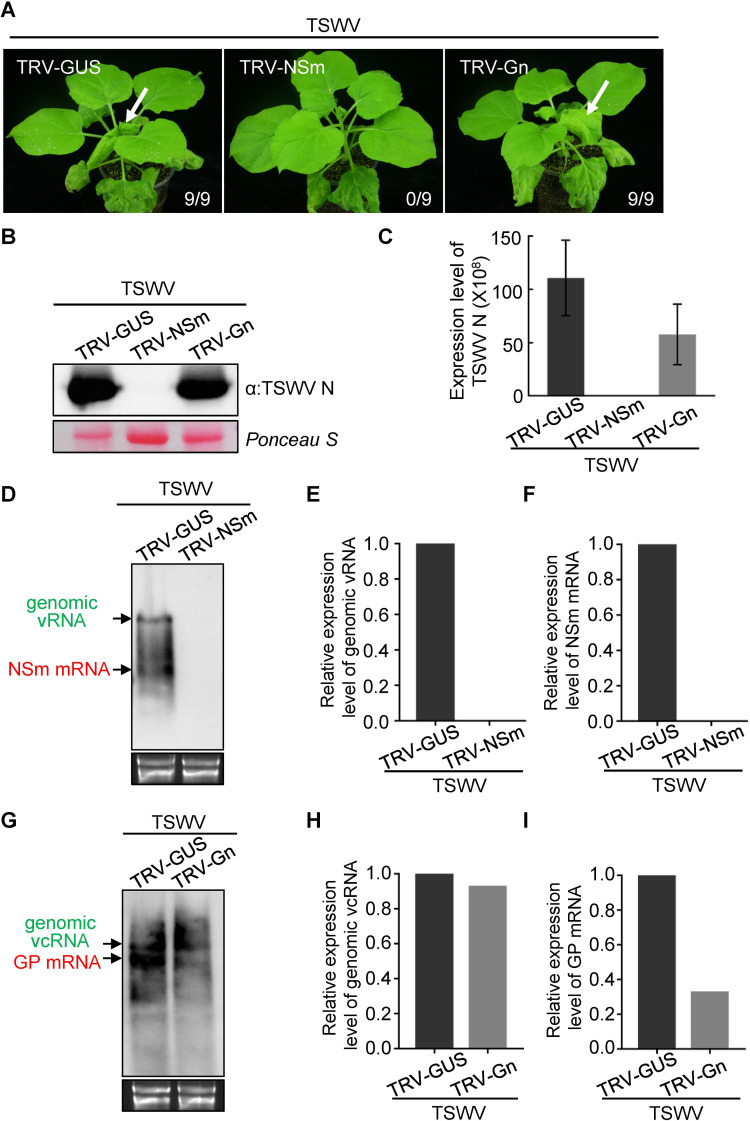
TRV-induced RISC mainly targets viral mRNA but not genomic RNA of TSWV M segment *in planta*. (A) The TRV-GUS, TRV-NSm and TRV-Gn constructs were used to infect *N*. *benthamiana* plants through agro-infiltration, respectively. At 10 days post agroinfitration, the leaves of TRV-GUS, TRV-NSm and TRV-Gn pre-infected plants were challenged with TSWV inoculum. The phenotype of *N*. *benthamiana* plants was monitored and photographed at 9 days post TSWV inoculation. The white arrow indicates the typical TSWV symptoms in systemically-infected leaves of TRV-GUS and TRV-Gn pre-infected *N*. *benthamiana* plants. The numbers showing in the image are the total number of the inoculated plants verses the infected plants observed from the different treatments. (B) and (C) Detection of TSWV accumulation in systemically-infected leaves of TRV-NSm, TRV-Gn and TRV-GUS pre-infected *N*. *benthamiana* plant by western blot and qRT-PCR analysis, respectively. TSWV N-specific antibody and TSWV-N specific primers were used to detect accumulation of TSWV N protein and viral RNAs, respectively. The Ponceau S stained gel was used to show the protein loading for samples. Error bars represent SD (n = 3). (D) Northern blot analysis of genomic vRNA and NSm mRNA of TSWV M segment targeted by pre-assembled RISC from TRV-GUS and TRV-NSm in *N*. *benthamiana* plants using a strand-specific DIG-labelled NSm probe. Samples were collected at 3 dpi from TSWV inoculated leaves of TRV-GUS and TRV-NSm treated plants. Genomic vRNA and viral NSm mRNA bands are indicated by the arrows in green and red, respectively. Ethidium bromide staining was used to show RNA loading. (E) and (F) Quantification of the accumulation level of genomic vRNA and NSm mRNA, respectively, shown in Fig 4D. (G) Northern blot analysis of genomic vcRNA and GP mRNA of TSWV M segment targeted by pre-assembled RISC from TRV-GUS and TRV-Gn in *N*. *benthamiana* plants using a strand-specific DIG-labelled Gn antisense probe. Samples were collected at 3 dpi from TSWV inoculated leaves of TRV-GUS and TRV-Gn pre-infected plants. Genomic vcRNA and GP mRNA bands are indicated by the arrows in green and red, respectively. Ethidium bromide staining was used to show RNA loading. (H) and (I) Quantification of the expression level of genomic vcRNA and GP mRNA, respectively, shown in Fig 4G. All experiments were repeated more than three times.

In analogy to the approach with TRV-N and TRV-NSs, antiviral RISC was tested on the ambisense encoded genes from the TSWV M RNA, *i*.*e*. NSm and GP (Fig 1A). Whereas the viral glycoproteins processed from GP are dispensable for the infection cycle in plants [28,49], NSm is essential for cell-to-cell and long-distance movement and its silencing would lead to a subliminal infection, *i*.*e*. restrict the infection to the initially infected cell [28,29,45]. For each gene a 300 bp gene fragment was cloned into TRV, rendering TRV-NSm and TRV-Gn and next used to pre-infect *N*. *benthamiana* plants via agro-infiltration, using TRV-GUS as negative control (S3 Fig). At 10 days post agro-infiltration of TRV-NSm, TRV-Gn and TRV-GUS, the local (lower) leaves were challenged with TSWV. Typical TSWV symptoms were detected in the (upper) systemically-infected leaves of TRV-GUS or TRV-Gn pre-treated plants about 9 days post TSWV inoculation (Fig 4A). However, no viral symptoms were observed in the systemic leaves of plants pre-treated with TRV-NSm. Upon analyses of systemically-infected leaves using western blot and qRT-PCR, N protein and viral RNA was detected in TRV-GUS and TRV-Gn pre-treated plants but not in TRV-NSm pre-treated plants (Fig 4B and 4C). When the TSWV inoculated leaves were analyzed by northern blots using a strand specific probe for NSm, both genomic M vRNA and NSm mRNA were detected in the pre-treated TRV-GUS plants but not in those that were pre-treated with TRV-NSm (Fig 4D–4F). In the case a strand specific probe for Gn was used, genomic M vcRNA and GP mRNA were detected in the TSWV inoculated leaves of both TRV-Gn and TRV-GUS (control) pre-treated plants, however the accumulation of GP mRNA relative to the genomic RNA molecules in TRV-Gn pre-treated plant was significantly lower (Fig 4G–4I). Similar results were obtained when plants were challenged with TSWV 15 days after the TRV-GUS, TRV-NSm or TRV-Gn treatment of *N*. *benthamiana* (S4C and S4D Fig). Altogether, these data suggested that RISC, the core effector of RNA silencing, mainly targets tospoviral mRNA.

### Purified RISC of a Euro-Asia type *tospovirus* targets naked genomic RNA *in vitro* but not when embedded into viral RNPs

**Fig 5:**
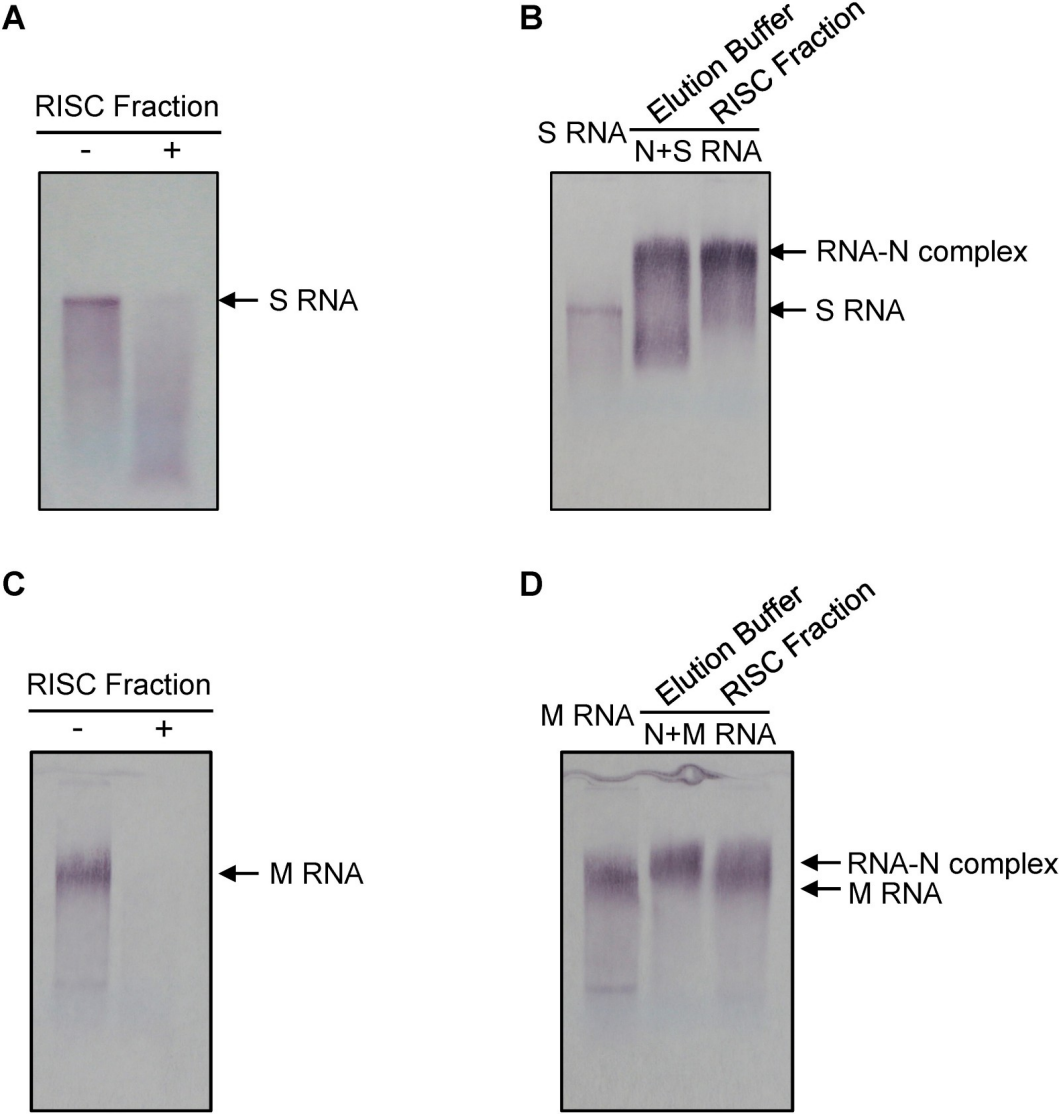
RISC fraction from TZSV-infected plant targets naked genomic RNA but not the genomic RNA complexed with N proteins *in vitro*. (A) and (C) *In intro* cleavage assay of naked genomic RNA of TZSV S and M segment by isolated RISC chromatography fractions. Chromatography fraction 5 (S6 Fig) from TZSV-infected *N*. *benthamiana* were incubated with DIG-labeled *in vitro* full-length RNA transcripts of TZSV S (100 ng; A) and M (100 ng; B) segment, respectively. (B) and (D) *In vitro* cleavage assay of TZSV genomic S or M RNA complexed with N protein by the RISC fraction. DIG-labelled full-length S (B) or M (D) genomic RNA was incubated with TZSV N protein to form N-RNA complexes. RISC fraction 5 isolated from TZSV-infected plants was added to N-S RNA or N-M RNA complexes. The blots were developed by AP-labeled anti-digoxigenin antibodies and followed with BCIP/NBT staining.

To further substantiate the above observations, experiments were repeated but now using *Tomato zonate spot virus* (TZSV), a distinct representative of the European-Asian type tospoviruses. In analogy to TSWV, RISC was isolated from TZSV-infected leaves using Hydroxyapatite and Superdex S-200 HR gel column chromatography. Pilot experiments revealed that gel filtration fractions 2–7 contained RISC associated ribonuclease activity (S5 Fig). Fraction 5 exhibited high RSIC nuclease activity and was next incubated with DIG-labelled full-length S RNA transcripts of TZSV. The results showed that the addition of the anti-TZSV RISC fraction completely digested TZSV S RNA (Fig 5A). When prior to the incubation with the RISC fraction, the DIG-labelled full-length S RNA transcripts were first incubated with TZSV N protein to form N-RNA complexes *in vitro*, the addition of the TZSV RISC fraction failed to degrade the S RNA (Fig 5B). Similar results were obtained when naked DIG-labelled full-length genomic M RNA transcripts of TZSV or complexed with N protein were incubated in the presence of the anti-TZSV RISC fraction (Fig 5C and 5D). These results indicated that TZSV RNPs, like with TSWV, well protect viral genomic RNA against cleavage by antiviral RISC *in vitro*.

### TRV-induced RISC activity against TZSV mainly targets viral mRNA but not genomic RNA of TZSV *in planta*

**Fig 6:**
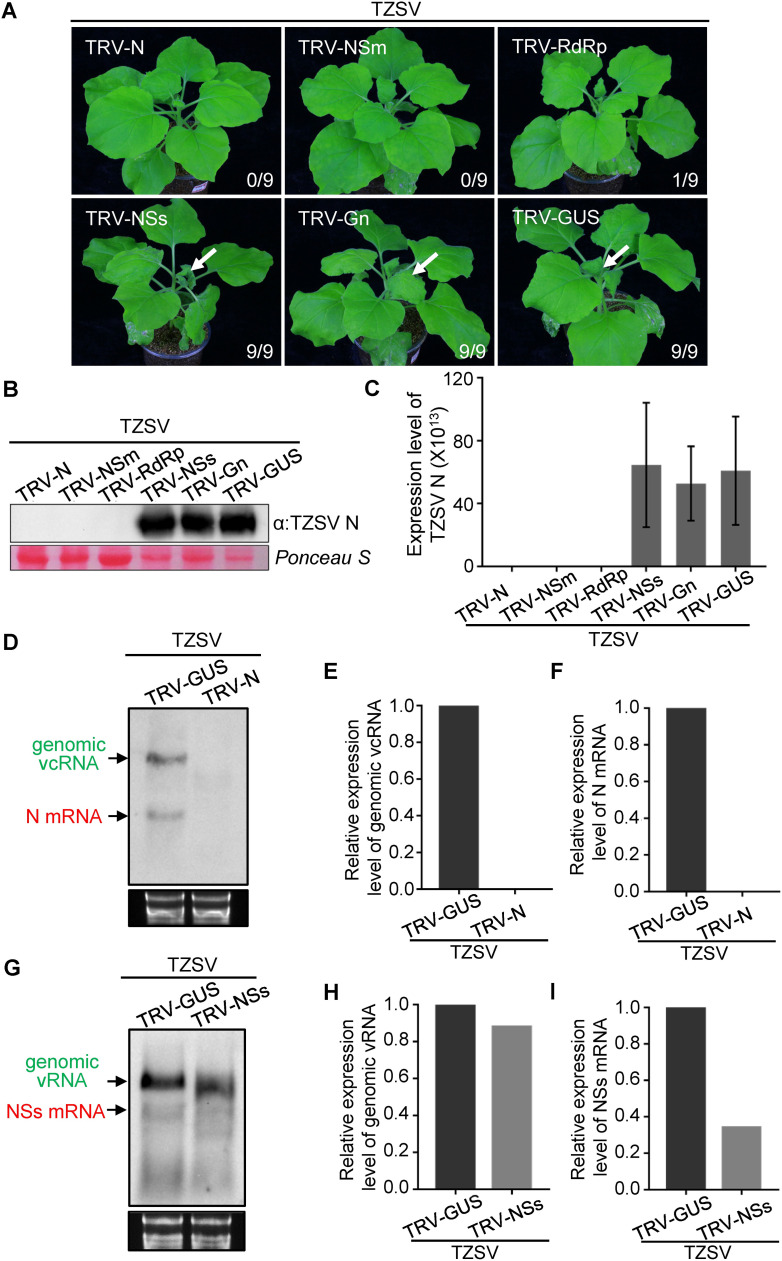
TRV-induced RISC mainly targets viral mRNA but not genomic RNA of TZSV *in planta*. (A) TRV-N, TRV-NSs, TRV-NSm, TRV-Gn, TRV-RdRp and TRV-GUS were used to pre-infect *N*. *benthamiana* plants via agro-infiltration, respectively. At 10 days post agroinfitration, the infiltrated leaves of TRV pre-infected plants were challenged with TZSV inoculum. The phenotype of *N*. *benthamiana* plants challenged by TZSV was monitored and photographed at 9 days post TZSV inoculation. The white arrow indicates the typical TZSV symptoms in systemically-infected leaves of TRV-NSs, TRV-Gn and TRV-GUS pre-infected *N*. *benthamiana* plants. The numbers showing in the image are the total number of the inoculated plants versus the infected plants observed from the various treatments. (B) and (C) Detection of TZSV accumulation in systemically-infected leaves of TRV-N, TRV-NSs, TRV-NSm, TRV-GP, TRV-RdRp and TRV-GUS pre-infected *N*. *benthamiana* plant by western blot and qRT-PCR analysis, respectively. TZSV N-specific antibody was used to detect accumulation of TSWV N protein on western blot. The Ponceau S stained gel shows the equal protein loading of samples. TZSV-N specific primers were used to detect viral RNAs by qRT-PCR. Error bars represent SD (n = 3). (D) Detection of genomic vcRNA and N mRNA of TZSV S segment targeted by antiviral RISC from TRV-N, TRV-NSs and TRV-GUS pre-infected plant by northern blot. Samples were collected at 3 dpi from the TZSV inoculated leaves of TRV pre-infected plants. Genomic vcRNA and viral mRNA bands are indicated by the arrows in green and red, respectively. Ethidium bromide staining was used to show equal RNA loading. (E) and (F) Quantification of the expression level of genomic vcRNA and N mRNA, respectively, shown in Fig 6D. (G) Northern blot analysis of viral genomic vRNA and NSs mRNA of TZSV S segment targeted by pre-assembled RISC from TRV-GUS and TRV-NSs in *N*. *benthamiana* plants using strand specific DIG-labelled NSs probe. Samples were collected at 3 dpi from TZSV inoculated leaves of TRV-GUS and TRV-NSs pre-infected plants. Genomic vRNA and viral NSs mRNA bands are indicated by the arrows in green and red, respectively. Ethidium bromide staining was used to show equal RNA loading. (H) and (I) Quantification of the accumulation level of viral genomic vRNA and NSs mRNA, respectively, shown in Fig 6G.

To analyze RISC activity against TZSV *in planta*, a similar approach was applied as described for TSWV. To this end, a 300 bp gene fragment of TZSV *N*, *NSs*, *NSm*, *GP* and *RdRp* was cloned into a TRV vector, rendering TRV-N, TRV-NSs, TRV-NSm, TRV-GP and TRV-RdRp, respectively. Next, these clones, including TRV-GUS as negative control, were used to pre-infect *N*. *benthamiana* plants by agro-infiltration to induce anti-TZSV gene-specific RISC complexes. At 10 days post *agro-*infiltration, the local leaves of TRV pre-infected plants were inoculated with TZSV. About 9 days after TZSV inoculation typical TZSV symptom were observed in systemically infected leaves of TRV-GUS, TRV-NSs and TRV-Gn pre-treated plants (Fig 6A, arrows). However, no viral symptoms were discerned in systemic leaves of TRV-N, TRV-NSm and TRV-RdRp pre-treated plants (Fig 6A). Western blot and qRT-PCR analyses confirmed the presence of TZSV in the systemically infected leaves of TRV-GUS, TRV-NSs, TRV-Gn pre-infected plants but not in those of TRV-NSm, TRV-N and TRV-RdRp pre-infected plants (Fig 6B and 6C). To further determine the RNA targets of TRV-induced anti-TZSV RISC *in planta*, total RNA was extracted from the TZSV inoculated leaves of TRV-GUS, TRV-NSs and TRV-N pre-infected plants and analyzed by northern blot using strand specific probes to the respective viral genes. Similar to the results earlier obtained with TSWV *in planta*, TRV-GUS plants showed the presence of TZSV genomic S vcRNA and N mRNA, but these were absent from TRV-N pre-treated plants (Fig 6D and 6F). TZSV genomic S vRNA and NSs mRNA were still detected in plants pre-treated with TRV-NSs and the TRV-GUS control, however levels of NSs mRNA were significantly reduced in those of TRV-NSs (Fig 6G–6I).

### The antiviral RISC targets (sub)genomic (m)RNA of CMV, a representative plant positive-sense RNA virus

We next investigated the targets of antiviral RISC to *Cucumber mosaic virus* (CMV), a representative ss(+)RNA plant virus. CMV contains a tripartite RNA genome of which RNA2 encodes two proteins, 2a and 2b (S6 Fig). While CMV 2a is expressed from the genomic RNA 2 and is essential for viral replication [50], CMV 2b is expressed from a subgenomic mRNA molecule, and dispensable for viral replication. CMV 2b mutants still are able to infect *N*. *benthamiana* but are attenuated in their symptomatology [51,52]. In analogy to the strategy applied to TSWV and TZSV, TRV carrying CMV *2a* and *2b* gene fragments were generated to induce anti-CMV RISC in *N*. *benthamiana*. We hypothesized that if anti-2a and anti-2b activated RISC would both target CMV genomic RNA 2, no CMV infection would be established. If, alternatively, anti-2b activated RISC would only target and silence subgenomic mRNA 2b, CMV infection would not be abrogated.

**Fig 7:**
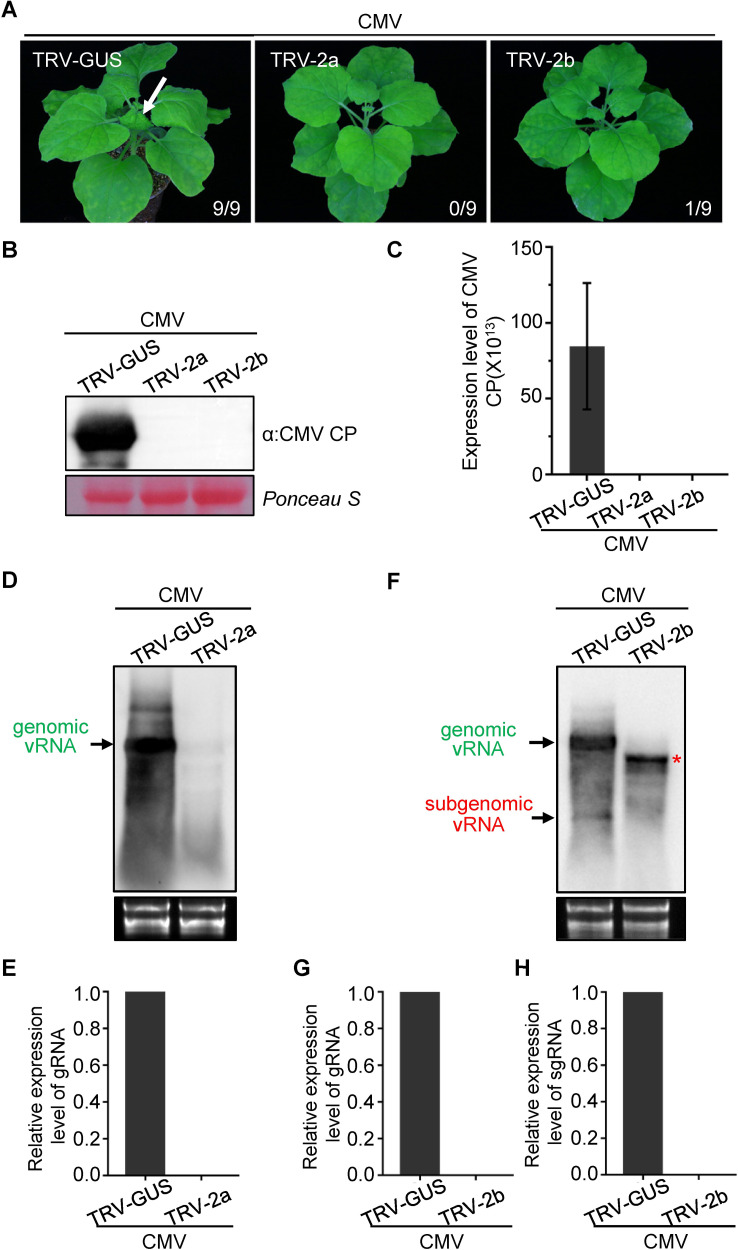
TRV-induced RISC targets genomic RNA and subgenomic mRNA of CMV *in planta*. (A) TRV-2a, TRV-2b and TRV-GUS constructs were used to pre-infect *N*. *benthamiana* plants by agro-infiltration, respectively. At 10 days post agroinfitration, the leaves of TRV-2a, TRV-2b and TRV-GUS pre-infected plants were rub inoculated with sap of CMV-Fny from freshly-infected leaf tissues. The phenotype of *N*. *benthamiana* plants was photographed at 12 days post CMV inoculation. The white arrow indicates the typical CMV symptoms in systemically-infected leaves of TRV-GUS pre-infected *N*. *benthamiana* plants. The numbers showing in the image are the total number of the inoculated plants versus the infected plants observed from the various treatments. (B) and (C) Detection of CMV accumulation in systemically-infected leaves of TRV-2a, TRV-2b and TRV-GUS pre-infected *N*. *benthamiana* plants by western blot and qRT-PCR analysis, respectively. CMV-CP specific antibody was used to detect the accumulation of CMV on western blot. The Ponceau S stained gel was used to show the equal protein loading for samples. CMV CP specific primers were used to detect viral RNAs in qRT-PCR. Error bars represent SD (n = 3). (D) Northern blot analysis of genomic(g) RNA2 targeted by pre-assembled RISC from TRV-2a in *N*. *benthamiana* plants using a strand specific DIG-labeled CMV 2a probe. Samples were collected at 3 dpi from the CMV inoculated leaves of TRV-GUS and TRV-2a pre-infected plants. The band of genomic viral (v) strand RNA is indicated by the arrow in green. Ethidium bromide staining was used to show RNA loading. (E) Quantification of the expression level of genomic vRNA in Fig 7D. (F) Northern blot analysis of genomic vRNA2 and subgenomic (sg) RNA of CMV targeted by pre-assembled antiviral RISC from TRV-2b in *N*. *benthamiana* plants using strand-specific DIG-labeled CMV 2b probe. Samples were collected at 5 dpi from the CMV inoculated leaves of TRV-GUS and TRV-2b pre-infected plant. Ethidium bromide staining was used to check and show equal loading of RNA. A 190 bp fragment (143–333 nt) from the C-terminal region of 2b was used for the construction of TRV-2b and a 170 bp fragment (164–333 nt) from the C-terminal region of 2b was used as template to prepare the DIG-labeled probe. The red asterisk indicates the band of TRV RNA2 from TRV-2b infected plants detected by the strand-specific DIG-labeled CMV 2b probe. (G) and (H) Quantification of the expression level of genomic vRNA2 and sgRNA for 2b, respectively, shown in Fig 7F.

TRV clones were generated containing a 300 bp gene fragment of *2a* and a 200 bp fragment of *2b* of CMV and, next to TRV-GUS as control, used to pre-infect *N*. *benthamiana* plants by agro-infiltration. At 10 days post agro*-*infiltration, the local leaves of TRV pre-infected plants were inoculated with CMV-Fny. While typical CMV symptoms were observed in the systemically-infected leaves of TRV-GUS pre-infected plants 12 days post CMV inoculation, no viral symptoms were observed in any of the systemic leaves from TRV-2a or TRV-2b pre-treated plants (Fig 7A). Western blot and qRT-PCR analyses confirmed the absence of CMV from the systemic leaves of TRV-2a and TRV-2b pre-treated plants (Fig 7B and 7C). Using northern blot analysis CMV genomic vRNA2 was detected in TRV-GUS pre-infected plants using either 2a or 2b strand specific probes, while the strand specific probe for 2b additionally detected the subgenomic RNA2b (Fig 7D–7H). In plants pre-infected with TRV-2a or TRV-2b, neither genomic vRNA2 nor sub-genomic RNA2b were detected (Fig 7D–7H), indicating that for CMV the antiviral RISC is able to target viral (sub)genomic (m)RNA.

## Discussion

In plants RNA silencing is part of the host innate immunity system against viruses in which, during viral infection, RISC becomes programmed to specifically identify and degrade the invasive viral RNAs [5,6]. RISC typically targets single stranded RNA in a sequence-dependent manner, by a siRNA guide strand loaded into the RISC complex. Unlike with (+)ssRNA plant viruses, genomic and antigenomic RNAs of plant NSVs are single-stranded and tightly wrapped by N protein into RNPs complexes [19,20,47]. In this study, antiviral RISC activity was analyzed against plant NSVs, to investigate whether antiviral RISC is able to target NSV genomic RNA complexed into RNPs. To this end, fractions containing (antiviral) RISC associated nuclease activity were isolated from *N*. *benthamiana* as previously described for TRV- or *Tombusvirus-* infected plants [12–14], and biochemically analyzed *in vitro* on the targeting of viral genomic RNA, either naked or complexed with N protein. In addition, and to substantiate the *in vitro* findings, *in planta* studies were performed in which antiviral RISC was induced by a pre-infection with TRV containing (partial) gene sequences from the NSV tospoviruses TSWV or TZSV or the (+)ssRNA CMV. The results showed that whereas for CMV all genomic and subgenomic (m)RNA molecules were targeted by RISC, RISC primarily targets viral mRNA from the NSV tospoviruses *in vitro* and *vivo*. Genomic (v and vc) RNA complexed with N *in vitro*, or within RNPs from a natural infection were hardly or not targeted. Altogether, the findings indicate that antiviral RNAi primarily targets tospoviral mRNAs whilst their genomic RNA molecules are well protected in RNPs against RISC-mediated cleavage. Considering the important role of RNPs in the replication cycle of all NSVs, the observations made in this study are likely applicable to all viruses belonging to this group. Whether, in addition, the formation of RNPs also presents a strategy of the virus to evade from antiviral RNAi remains to be debated.

The sequence specific degradation by antiviral RISC was nicely demonstrated in this study as well, where purified antiviral RISC fractions from tospovirus infected *N*. *benthamiana* did not target/degrade GFP RNA, while corresponding fractions isolated from mock-treated *N*. *benthamiana* did not cleave tospoviral RNA. Previous studies have reported on two different RISC complexes in *Arabidopsis thaliana*. One complex containing an AGO1 core and sized >500 kDa, and another complex containing AGO4 and sized 60–200 kDa [53]. During the purification of RISC complexes from TSWV-infected *N*. *benthamiana* plants two different peak fractions (fractions 4–9 and fractions 12–16) were also observed to exhibit antiviral RISC-activity. However, and in contrast, only one (fractions 2–7) was isolated from TZSV-infected *N*. *benthamiana*. RISC fractions isolated from TRV-infected *N*. *benthamiana* plants also distributed in two different peaks [13], while no fractions isolated from wild type tobamovirus infected *N*. *benthamiana* plant contained RISC-activity. Earlier studies on tombusviruses showed that the RISC-activity could only be detected in fractions collected from TBSV P19 mutant-infected plants but not from wild type virus infected plants [12]. It is not unlikely that all these differences relate to different antiviral RNAi and viral counter defense strategies, although the differences observed between TSWV and TZSV tospoviruses remain a bit puzzling.

Previous studies have shown that tospovirus-specific siRNAs (vsiRNAs) from all three RNA segments are generated during an infection of *N*. *benthamiana* and tomato [54,55]. Considering that the genomic RNA strands are well embedded and protected in RNPs against RISC cleavage, tospovirus siRNAs produced likely result from dsRNA precursor structures that originate from the “naked” viral mRNAs. In light of this, the wide distribution of vsiRNAs all over the genome makes sense, since tospoviral mRNA sequences almost span the entire genome. Consistent with the finding that viral mRNA but not genomic (v and vc) RNA is targeted, siRNAs are rarely detected from the intergenic region of S RNA and M RNA segments [54,55]. Although part of the intergenic region is still reflected in the M- and S-RNA transcribed subgenomic mRNAs, and fold into hairpin structures that function in the termination of viral mRNA transcription, they have been proposed to be associated with NSs and/or elements from the translation machinery to engage in translation, which would also protect them against antiviral RNAi targeting.

Findings from this study are also in agreement with earlier studies on pathogen-derived resistance engineered against TSWV [56]. TRV-induced RISC-activation against NSs or GP, both dispensable for viral replication, did not confer resistance against tospoviruses. Genomic RNA replication was still detected and indicated that these strands are not targeted by RISC. This observation is consistent with earlier studies in which pre-infection of apple latent spherical virus carrying NSs gene fragments also did not protect against three different tospoviruses [57]. On the other hand, targeting of N and NSm prevented TSWV infection and is easily explained from their important roles in the life cycle. RISC targeting of only N mRNA would deplete cells from N protein production and abrogate viral replication, while targeting NSm RNA would prevent production of viral movement protein and block local and systemic spread of the virus. Earlier reports have also shown that transgenic expression of N or NSm gene sequences protects against tospovirus infections [56,58]. While transgenic tobacco expressing NSm are immune to TSWV infection at the plant level, protoplasts isolated from these transformants still support replication of TSWV [58], providing further support for the idea that only the (NSm) transcripts, and not the RNPs, are being targeted by RISC, allowing replication to continue but limited to the infected cell.

Studies from other plant NSVs provide support for the idea that antiviral RISC targeting of tospovirus mRNAs, but not RNPs, widely applies to other/all plant NSVs, Rice stripe virus is a tenuivirus from the *Phenuiviridae* within the order *Bunyavidales*. Transgenic rice plants that express (partial) sequences of the *C3* gene, coding for the nucleocapsid protein, or the *C4* gene, encoding the viral movement protein, protect against RSV infection, similarly as observed with tospoviruses. On the other hand, transgenic plants expressing (partial) sequences of *p3*, encoding a suppressor of RNA silencing, or *p4*, encoding a non-structural protein of unknown function, did not provide resistance to RSV infection [59]. Proteins p3 and pC3 are encoded in ambisense by the RNA3 segment of RSV, whereas p4 and pC4 genes are encoded in ambisense by the RNA4 segment of RSV. Being encoded from the same RNA segment, their individual silencing and different outcomes on resistance, provides further support for the idea that viral mRNA, but not the genomic (v and vc) RNA present in RNPs, is the primary target of host antiviral RISC. Defense and counter-defense is an endless arms race between plant host and virus. Whether the embedding of genomic (v and vc) RNAs into viral RNPs should/can be regarded as another weapon from the virus to protect against antiviral RISC remains a matter of debate.

**Fig 8:**
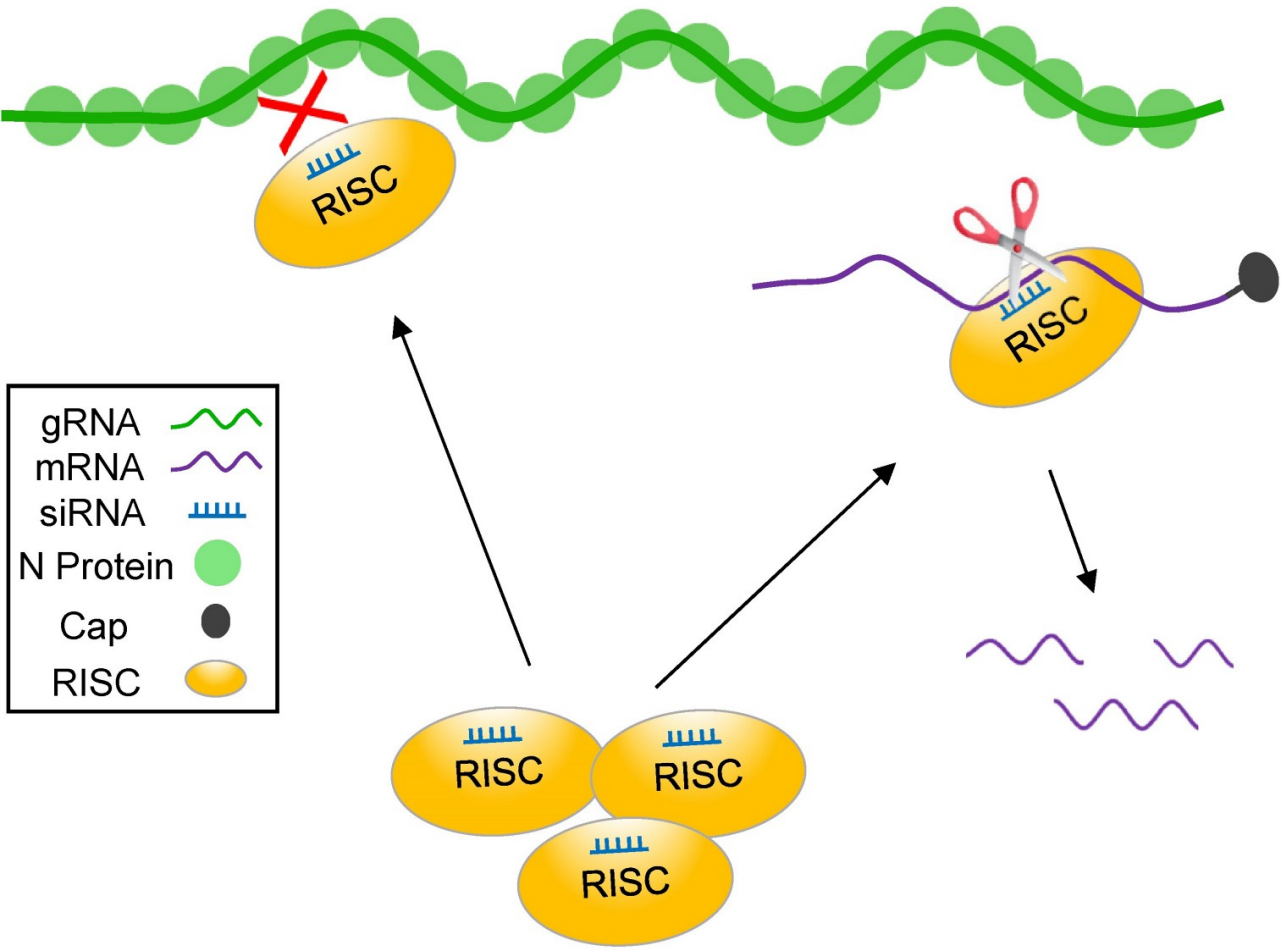
Model on antiviral RISC targeting of tospoviruses, a representative segmented plant NSVs: antiviral RISC mainly targets viral mRNA but not genomic RNA. Upon tospovirus infection of plants, host antiviral RISC effector complexes become activated. The viral (anti)genomic RNA of tospoviruses associates with N protein and assembles into RNP complexes that are resistant to host antiviral RISC. Viral mRNAs do not assemble into RNPs and are fully accessible for vsiRNA-mediated degradation by the Argonaute slicer activity of RISC.

In summary, using the segmented NSV tospovirus as model system, we have shown that RISC mainly targets viral mRNA and not genomic RNA. Fig 8 presents an overview summarizing the action of antiviral RISC against discriminate tospovirus RNA molecules produced during an infection cycle. Studies from other plant NSVs provide support for the idea that this is generic for all NSVs.

## Materials and method

### Plasmid construction

To generate TRV constructs carrying TSWV gene fragments, a 300 bp gene fragment from the N-terminal region of TSWV *N*, TSWV *NSs*, TSWV *Gn* and TSWV *NSm* was amplified, respectively, from the cDNA that was reverse transcribed from the purified total RNA of *N*. *benthamiana* leaves infected with TSWV lettuce isolate, then inserted into vector pTRV2 (YL156) to generate pTRV2-TSWV N, pTRV2-TSWV NSs, pTRV2-TSWV Gn and pTRV2-TSWV NSm.

To generate TRV constructs carrying TZSV gene fragments, a 300 bp fragment from the N-terminal region of TZSV *N*, TZSV *NSs*, TZSV *Gn*, TZSV *NSm* and TZSV *RdRp* genes was amplified, respectively, from the cDNA that was reverse transcribed from total RNA of *N*. *benthamiana* leaves infected with TZSV, then inserted into vector pTRV2 (YL156) to generate pTRV2-TZSV N, pTRV2-TZSV NSs, pTRV2-TZSV Gn, pTRV2-TZSV NSm and pTRV2-TZSV RdRp.

For generating the constructs pTRV2-CMV 2a and pTRV2-CMV 2b, a 300 bp gene fragment from the N-terminal region of *2a* and a 190 bp fragment from C-terminal region of *2b* was amplified from cDNA of CMV Fny, then inserted into pTRV2 (YL156).

Generation of constructs pET28a-TSWV N and the expression and subsequent purification of N protein has been described previously [39]. To generate pET28a-TZSV N, the coding region of the *N* protein gene was amplified from the cDNA of TZSV, inserted into downstream of the 6x His tag in bacterial expression vector pET28a digested with Nde I and EcoR I.

All primers of used in this paper are listed in S1 Table.

### Virus source and inoculation

Six-week old *N*. *benthamiana* plants were used in all agroinfiltration assays. The TSWV isolate from asparagus lettuce (TSWV-LE), TZSV isolate from tobacco and CMV Fny isolate were used in this study [60]. The TSWV, TZSV and CMV isolates were maintained in *N*. *benthamiana*. For long-term storage, infected new *N*. *benthamiana* leaves were kept in a -80°C. For plant inoculation, about 1 g fresh infected leaves of *N*. *benthamiana* were ground in 10 mL PB buffer (10 mM sodium phosphate, pH 7.0) and this crude extract was mechanically inoculated onto plant leaves. The virus-inoculated *N*. *benthamiana* plants were grown in a growth chamber at 25°C and 16 h light/8 h dark photoperiod.

### Hydroxyapatite column and Superdex S-200 column chromatography

The isolation of RISC fractions from virus infected plants was performed as described previously with slight modification [12–14]. Briefly, 40 g TSWV-infected, TZSV-infected or mock-inoculated *N*. *benthamiana* leaf tissue was homogenized in liquid nitrogen and the powder was resuspended in 40 mL loading buffer (10 mM sodium phosphate, pH 6.8). The homogenate was filtered through two layers of gauze and subsequently centrifuge for 15 min twice at 14,000 ×g at 4°C. Supernatant (40 mL) was loaded onto a 25×3.6 cm^2^ column packed with 50 mL Bio-Gel HT Hydroxyapatite (Bio-Rad, Hercules, CA). Chromatography separations were performed on ÄKTA avant 25 (GE Healthcare). After washing the column with 300 mL loading buffer, the bound proteins were eluted using a linear gradient of sodium phosphate buffer (pH 6.8) from 10–200 mM at a flow rate of 1.0 mL/min. Fractions were analyzed for the presence of active RISC by RNA cleavage assay and stored at -80°C. Active RISC fractions (0.5~1 mL) isolated from Hydroxyapatite column were further fractionated on Superdex 200 Increase 10/300 GL column (GE Hearlthcare, UK) pre-equilibrated with elution buffer (10 mM sodium phosphate, pH 6.8) at a flow rate of 0.5 mL/min. Every fraction (600 μL) was analyzed for RNA cleavage activity and active RISC fractions were stored at -80°C until further use.

### Preparation of RNA transcripts by *in vitro* transcription, protein expression and purification

RNA transcripts were generated using the T7 Riboprobe *in vitro* Transcription kit (Promega, Madison, WI). DNA templates was prepared by directly amplifying the full length TSWV S, TSWV M, TZSV S and TZSV M from full length cDNA clones of TSWV and TZSV [45]. The T7 promoter was added into each forward primer so as to generate PCR products containing a T7 promoter. RNA transcripts were synthesized in a reaction mixture containing 10 mM ATP, CTP, GTP (each), 6.5 mM UTP and 3.5 mM DIG-11-UTP (Roche, Germany), 10X T7 RNA Polymerase Buffer (Takara, Japan), 500 ng PCR amplified products as the template (containing a 5’ T7 promoter sequence), 20 units of RNase inhibitor, and 40 units of T7 polymerase and incubated at 37°C for 3 h. The concentrations of RNA transcripts were determined by a NanoDrop spectrophotometer (Thermo Scientific).

Protein expression and purification was performed as described [39]. Constructs pET28a-TSWV N, pET28a-TSWV N^R94A/R95A^ and pET28a-TZSV N were individually transformed into *Escherichia coli* strain Rosetta (DE3). To express the recombinant proteins, a 10 mL of overnight culture was transferred to a 1-liter culture and incubated at 37°C in a shaker incubator. When the optical density of culture at OD_600_ reached to 0.6, 0.1 mM isopropyl b-D-thiogalactopyranoside was added and incubated overnight at 20°C to induce protein expression. The purification of 6xHis-tag recombinant proteins was done using nickelnitrilotriacetic acid resin (Ni-NTA, Qiagen, Hilden, Germany) as described [39].

### RNA cleavage assays

RNA cleavage reactions were performed by incubating DIG-11-UTP-labeled RNA transcripts or DIG-labeled RNA complexed with N protein fractions from tospovirus-infected plants as described [12]. Five μg N proteins were mixed with 2 μg of DIG-labeled RNA probe in 10 μL volume and the mixtures were incubated for 10 min on ice to form N-RNA complexes. One μL of N-RNA mixtures was taken to incubate with 8 μL chromatography fractions. The mixtures were incubated for 10 min at room temperature to form N-RNA complexes. Cleavage reactions were incubated at 24°C for 60 minutes in presence of 15 U/mL RNase A inhibitor (TaKaRa, Japan) and 20 mM MgCl_2_. Control reactions were performed with elution buffer or with chromatography fractions from mock-inoculated plants. DIG-labeled RNA transcripts were resolved by electrophoresis on a 1% agarose gel and transferred to a Hybond-N+ membranes (GE Healthcare, UK) using the Bio-Rad semi-dry transfer unit (Bio-Rad, Hercules, CA). DIG-labelled signals on the blots were detected by AP-conjugated anti-digoxigenin antibodies and visualized by incubation in the presence of BCIP/NBT (Sangon Biotech, Shanghai, China) staining.

### *In planta* targeting of TSVW, TZSV and CMV by TRV-induced antiviral RISC

Agroinfiltration of *Tobacco rattle virus* (TRV) in *N*. *benthamiana* was performed as described previously [61,62]. Individual TRV constructs carrying tospovirus or CMV gene fragments were transformed into *Agrobacterium tumefaciens* by electroporation [63]. *Agrobacterium* carrying pTRV2-TSWV N, pTRV2-TSWV NSs, pTRV2-TSWV Gn, pTRV2-TSWV NSm, pTRV2-TZSV N, pTRV2-TZSV NSs, pTRV2-TZSV NSm, pTRV2-TZSV Gn, pTRV2-TZSV RdRp, pTRV2-CMV 2a, pTRV2-CMV 2b, or pTRV2-GUS control was equally mixed with *Agrobacterium* pTRV1 and adjusted the final concentration to OD_600_ = 0.25. After 2~4 hours incubation in the dark at room temperature, the mixtures were infiltrated into leaves of *Nicotiana benthamiana* plants to induce antiviral RISC formation to TSWV, TZSV and CMV. About 10 days after agroinfiltration, the infiltrated leaves of TRV-preinfected plants were mechanically inoculated with crude extract of TSWV, TZSV, and CMV, respectively, from freshly infected *N*. *benthamiana*. The phenotype of virus targeting by pre-assembled antiviral RISC derived from TRV constructs in *N*. *benthamiana* was monitored at 9–12 days post inoculation. The agroinfiltrated or virus-inoculated *N*. *benthamiana* plants were grown in a growth chamber at 22°C and 16 h light/8 h dark photoperiod.

### Western blot analysis

Protein levels of TSWV, TZSV and CMV in *N*. *benthamiana* were determined as described [64,65]. Total protein was extracted from systemically-infected leaf tissues of *N*. *benthamiana* at 9–12 days post virus inoculation. About 1 g leaf tissue was homogenized in 2 mL extraction buffer (10 mM sodium phosphate, pH 7.0) containing protease inhibitor cocktail (Sigma, Shanghai). Samples were separated on a 10% SDS-PAGE gel and transferred onto a polyvinylidene fluoride (PVDF) membrane (GE Healthcare, UK). To detect TSWV, TZSV and CMV accumulation, the blots were incubated with specific antibodies to TSWV N, TZSV N and CMV CP, followed with a secondary antibody containing HRP-conjugated goat anti-rabbit or goat anti-mouse. The blots were developed using the ECL Substrate Kit (Thermo Scientific, Hudson, NH, USA). ECL signals were visualized using the ChemiDoc Touch Imaging System (Bio-Rad). Blots were also stained with Ponceau S and used as reference for sample loading and quantification. Rabbit polyclonal antibodies specific for TSWV N and TZSV N were produced in this laboratory. Mouse Monoclonal antibody against CMV CP was kindly provided by Prof. Jianxiang Wu at Zhejiang University. HRP-conjugated goat anti-rabbit or goat anti-mouse IgG were purchased from Thermo Fisher Scientific (Invitrogen, Carlsbad, CA, United States).

### Quantitative RT-PCR

The RNA levels of TSWV, TZSV and CMV in *N*. *benthamiana* were determined using quantitative RT-PCR (qRT-PCR) [66]. Three pairs of primers for each gene were designed to detect and amplify the encoding regions of TSWV *N*, TZSV *N* and CMV *CP* (S1 Table). Quantitative RT-PCR amplifications were performed on a CFX Connect Real-Time System (Bio-Rad, California, U.S.A.) using a Hieff qPCR SYBR Green Master Mix (Yeasen, Shanghai, China). RNA transcripts of TSWV *N*, TZSV *N* or CMV *CP* were *in vitro* transcribed using linearized recombinant plasmids of pGEM-TSWV N, pGEM-TZSV N or pGEM-CMV CP and quantified using a NanoDrop spectrophotometer (Thermo Scientific). A series of ten-fold dilutions from 1 to 10^−5^ ng μL^−1^ of the RNA transcripts of TSWV N, TZSV N or CMV CP was prepared. The DNA amount of TSWV N, TZSV N or CMV CP in each dilution sample was represented as copies per μL, which was calculated using the following equation:

$$DNA\;(copies/\mu L)=\frac{\left(6.022\times 10^{23})\times (\frac{ng}{\mu L}\times 10^{-9}\right)}{DNA\;length\times 650}$$

The logarithm of the gene copy number in each dilution sample (x axis) was plotted against the corresponding crossing point value (y axis) to generate the standard curve for TSWV N, TZSV N and CMV CP. The linear regression equation, along with the coefficient of determination (R^2^), was used to evaluate the standard curves. After the cycle threshold (Ct) of TSWV, TZSV and CMV in infected N. *benthamiana* was determined, the copy numbers of TSWV N, TZSV N and CMV CP in infected plants were determined from the standard curves based on the crossing points of the samples. The expression of Elongation factor 1-alpha gene (NbEF1α, Niben101Scf08618g01001) was used as an internal control. Each experiment was performed with three biological replicates.

### Northern blot analysis

The genomic vRNAs, vcRNA and viral mRNA of TSWV, TZSV and CMV were detected by northern blot as described [45]. To analyze viral RNA on targeting by antiviral RISC induced via TRV carrying tospovirus or CMV gene fragments, total RNA was extracted at 3~5 dpi from TSWV-, TZSV- or CMV-inoculated leaves of *N*. *benthamiana* plants that were pre-infected with TRV constructs. Five to ten μg total RNA was resolved on a 1% agarose-formaldehyde gel and transferred to Hybond-N+ membranes (GE Healthcare, UK) [45]. A 300 bp gene fragment from the N-terminal region of TSWV and TZSV *N*, *NSs*, *Gn* and *NSm* was inserted into a TRV vector. To prepare the template for DIG-labeled probes that do not overlap with the gene fragment of TRV constructs, a 450 bp, 500 bp, 500 bp and 500 bp gene fragment from the C-terminal region of TSWV *N*, TSWV *NSs*, TSWV *GP* and TSWV *NSm*, respectively, was amplified from the full length cDNA clones of TSWV [45]; a 450 bp and 500 bp gene fragment from the C-terminal region of TZSV *N* and TZSV *NSs*, respectively, was amplified from TZSV cDNA; a 600 bp gene fragment from the C-terminal region of *2a* without overlapping with *2b*, and a 170 bp fragment from the C-terminal region of *2b* from cDNA clones of CMV-Fny. Due to the small size of CMV *2b*, the 2b probe overlapped with the *2b* fragment (190 bp) inserted in pTRV2-2b. The T7 promoter was added into each forward primer to generate PCR products containing a T7 promoter. The purified PCR products were used as DNA template for probes. Strand-specific DIG-labeled probes to detect for TSWV N, TSWV NSs, TSWV NSm, TSWV GP, TZSV N, TZSV NSs, CMV 2a and CMV 2b, respectively, were synthesized using a DIG High Prime RNA labeling kit (Roche, Basel, Switzerland). The blots were developed using a DIG-High Prime Detection Starter Kit II (Roche, Switzerland) according to the manufacturer’s instructions.

## Supporting information

S1 Fig RISC fractions isolated from tospovirus-infected plants specifically target naked genomic RNAs. (A) *In vitro* cleavage assay of naked genomic S RNA of TSWV by isolated chromatography fractions from TSWV infected *N*. *benthamiana*. Fractions from Superdex S-200 chromatography were incubated with digoxigenin (DIG) labelled *in vitro* full-length genomic S RNA of TSWV (100 ng). (B) Chromatography fractions isolated from mock-inoculated *N*. *benthamiana* plants were incubated with DIG-labeled full length genomic S RNA of TSWV (100 ng), and tested for their ability to cleave viral RNA. (C) Chromatography fractions isolated from TSWV-infected plants were incubated with DIG-labelled RNA transcript of GFP and tested on cleavage specificity.
(TIF)

S2 Fig N^R94A/R95A^ mutant was unable to protect viral genomic RNA from cleavage by host antiviral RISC. *In vitro* cleavage assay of TSWV genomic S RNA complexed with N and N^R94A/R95A^ mutant proteins by fraction 6 (S1A Fig) containing the RISC-activity. DIG-labelled full-length genomic S RNA was incubated with TSWV N protein and N^R94A/R95A^ to form N-RNA complexes. The RISC fraction was then added into N-RNA complexes to test the RNA protection by N and N^R94A/R95A^ protein. The signals on the blot were detected by AP-labeled anti-digoxigenin antibodies and followed with BCIP/NBT staining.
(TIF)

S3 Fig Diagram of pre-infection of TRV carrying TSWV gene fragments and followed with TSWV inoculation in *N*. *benthamiana*. Agrobacterium cultures containing pTRV2-NSs, pTRV2-N, pTRV2-NSm or pTRV2-Gn were equally mixed with Agrobacterium cultures containing pTRV1 and infiltrated into 4-leaf stage *N*. *benthamiana* plants using a 1 mL needle-less syringe. Ten days after agro-infiltration, these plants were rub inoculated with crude extracts from TSWV-infected leaves. The phenotype of TSWV challenged plants was monitored at 9 dpi.
(TIF)

S4 Fig TRV-induced RISC mainly targets viral mRNA but not genomic RNA of TSWV *in planta*. (A) Detecting genomic vRNA and NSs mRNA of TSWV S segment targeted by RISC from TRV-NSs and TRV-GUS plants using strand specific DIG-labelled NSs probe. (B) Northern blot analysis of viral genomic vcRNA and N mRNA of TSWV S segment targeted by pre-assembled RISC from TRV-GUS and TRV-N in *N*. *benthamiana* plants using strand specific DIG-labelled N probe. (C) Detection of genomic vRNA and NSm mRNA of TSWV M segment targeted by antiviral RISC from TRV-NSm and TRV-GUS pre-infected plants by northern blot analysis using strand specific DIG-labelled NSm probe. (D) Genome M vcRNA and GP mRNA were detected in TRV-Gn and TRV-GUS pre-infected plants using strand specific DIG-labelled Gn probe. *N*. *benthamiana* plants were agroinoculated with the TRV constructs and after about 15 days, to allow for pre-assembly of antiviral RISC, the local leaves were challenged with TSWV. Samples were collected at 5 dpi from the TSWV inoculated leaves of TRV pre-infected plants. Genomic vcRNA (or vRNA) and viral mRNA bands are indicated by the arrows in green and red, respectively. Ethidium bromide staining was used to show equal RNA loading of samples.
(TIF)

S5 Fig Isolation of chromatography fractions from TZSV-infected plants and *in vitro* cleavage assay of fractions using TZSV S RNA. Sixteen Superdex S-200 fractions were isolated from TZSV infected *N*. *benthamiana*. Every fraction (600 μL each) was incubated *in vitro* with digoxigenin (DIG) labeled full length S RNA of TZSV (100 ng) to examine which fraction contained the RISC associated nuclease activity to viral S RNA.
(TIF)

S6 Fig Genomic organization of CMV. CMV RNAs encodes five open reading frames 2a is encoded by genomic RNA2 of CMV. 2b is encoded by subgenomic RNA2 of CMV. The 1a, 2a and 3a ORFs expressed from the genomic strands are denoted in blue, whereas 2b and CP, expressed from a subgenomic mRNA, are denoted in red.
(TIF)

S1 Table Primers used in this study. (XLSX)
