# Supplementary figures and images for: Antiviral RISC mainly targets viral mRNA but not genomic RNA of tospovirus
Source: PLoS Pathog. 2021 Jul 28;17(7):e1009757. doi: 10.1371/journal.ppat.1009757 (PMC8351926; doi:10.1371/journal.ppat.1009757)

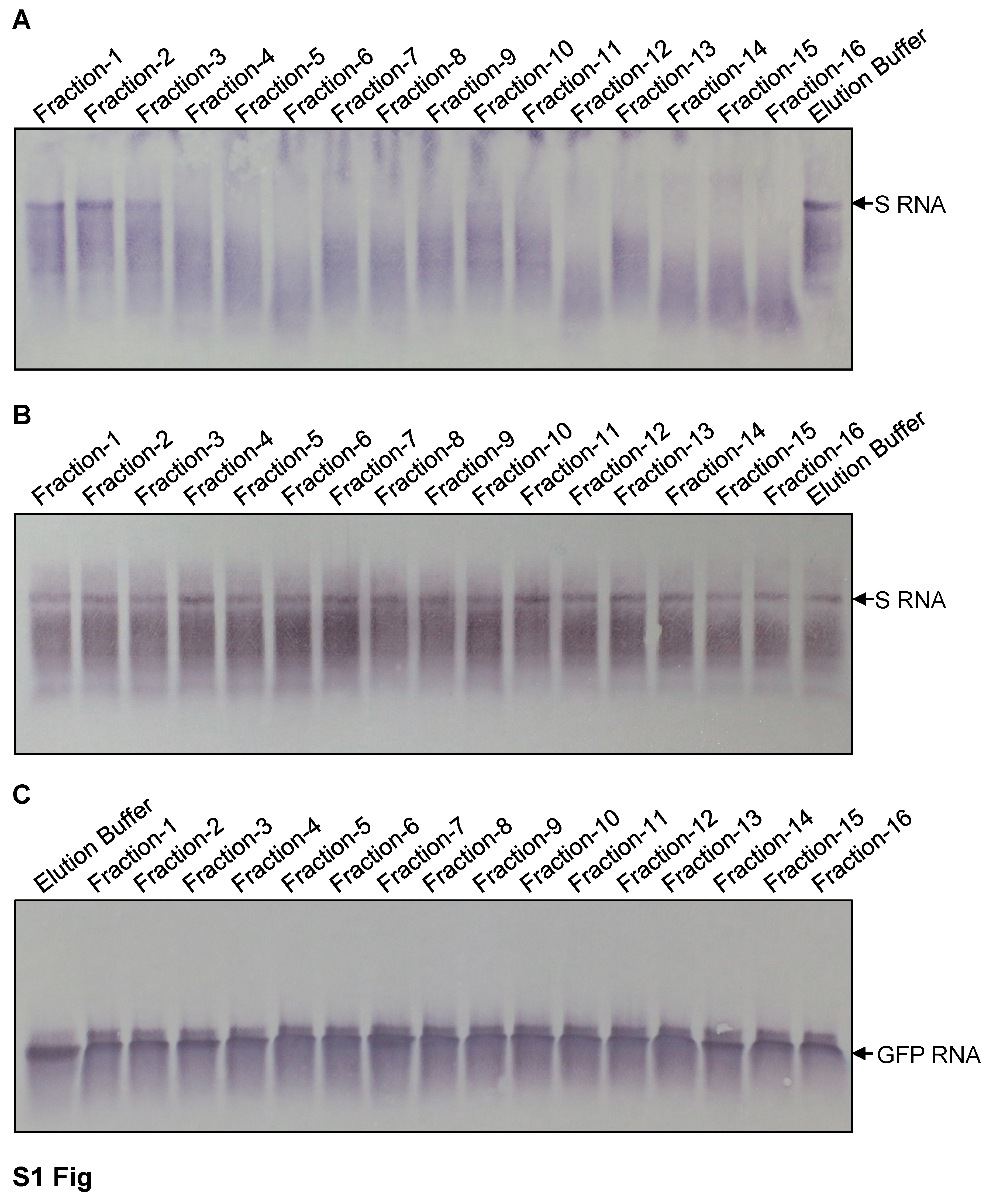

Supplement: S1 Fig — (A) In vitro cleavage assay of naked genomic S RNA of TSWV by isolated chromatography fractions from TSWV infected N. benthamiana. Fractions from Superdex S-200 chromatography were incubated with digoxigenin (DIG) labelled in vitro full-length genomic S RNA of TSWV (100 ng). (B) Chromatography fractions isolated from mock-inoculated N. benthamiana plants were incubated with DIG-labeled full length genomic S RNA of TSWV (100 ng), and tested for their ability to cleave viral RNA. (C) Chromatography fractions isolated from TSWV-infected plants were incubated with DIG-labelled RNA transcript of GFP and tested on cleavage specificity. (TIF) [file ppat.1009757.s001.tif]

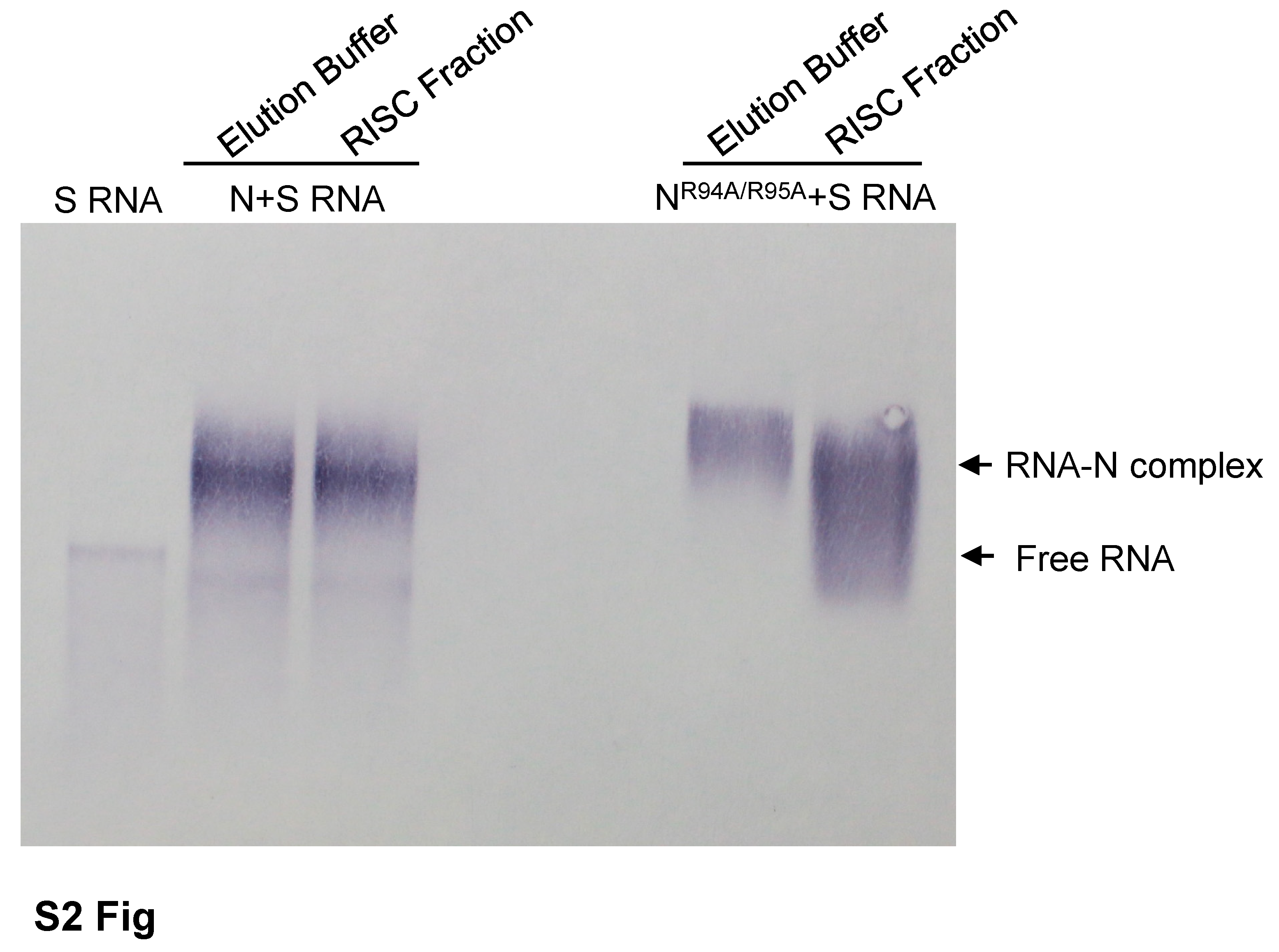

Supplement: S2 Fig — In vitro cleavage assay of TSWV genomic S RNA complexed with N and NR94A/R95A mutant proteins by fraction 6 (S1A Fig) containing the RISC-activity. DIG-labelled full-length genomic S RNA was incubated with TSWV N protein and NR94A/R95A to form N-RNA complexes. The RISC fraction was then added into N-RNA complexes to test the RNA protection by N and NR94A/R95A protein. The signals on the blot were detected by AP-labeled anti-digoxigenin antibodies and followed with BCIP/NBT staining. (TIF) [file ppat.1009757.s002.tif]

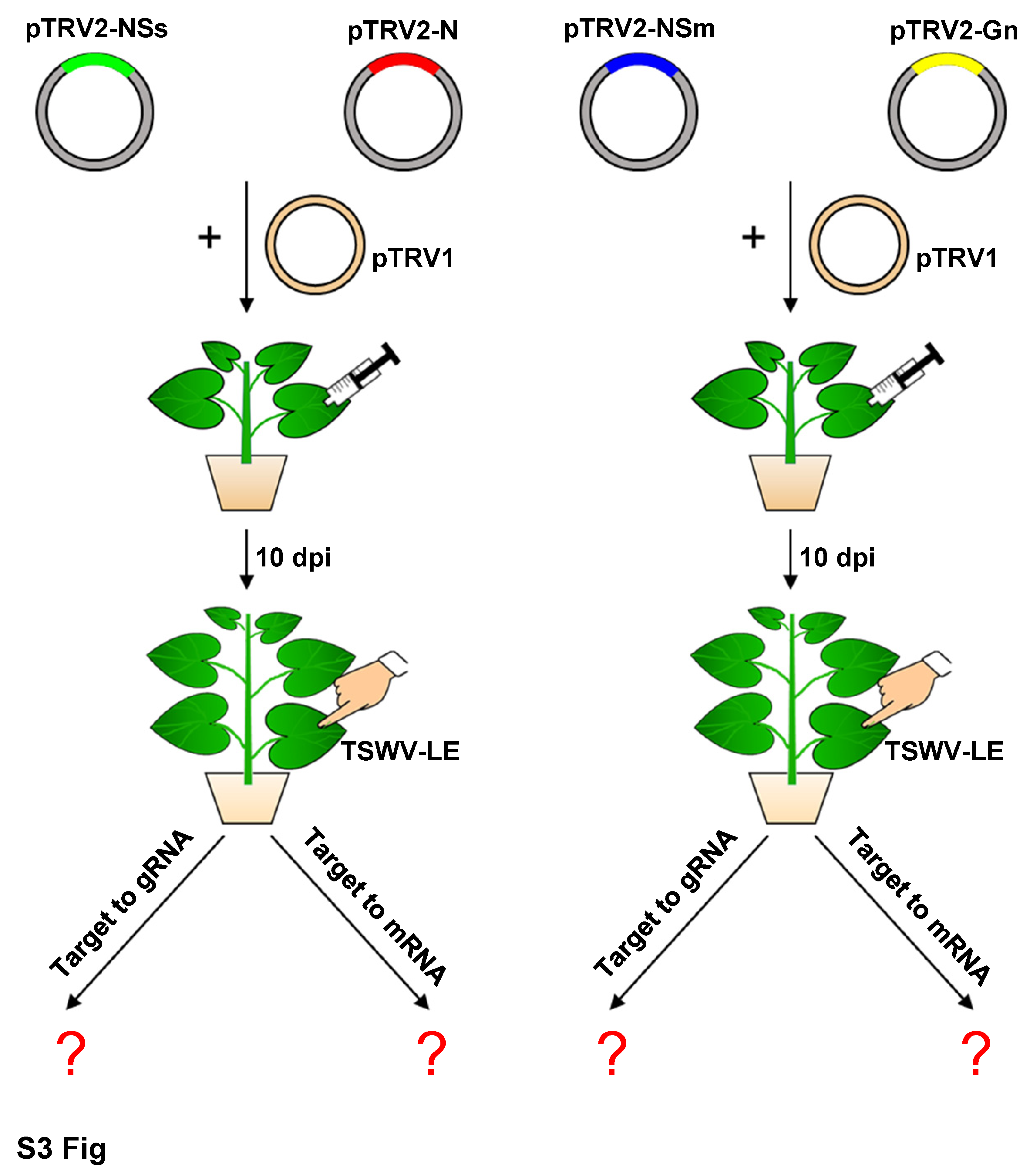

Supplement: S3 Fig — Agrobacterium cultures containing pTRV2-NSs, pTRV2-N, pTRV2-NSm or pTRV2-Gn were equally mixed with Agrobacterium cultures containing pTRV1 and infiltrated into 4-leaf stage N. benthamiana plants using a 1 mL needle-less syringe. Ten days after agro-infiltration, these plants were rub inoculated with crude extracts from TSWV-infected leaves. The phenotype of TSWV challenged plants was monitored at 9 dpi. (TIF) [file ppat.1009757.s003.tif]

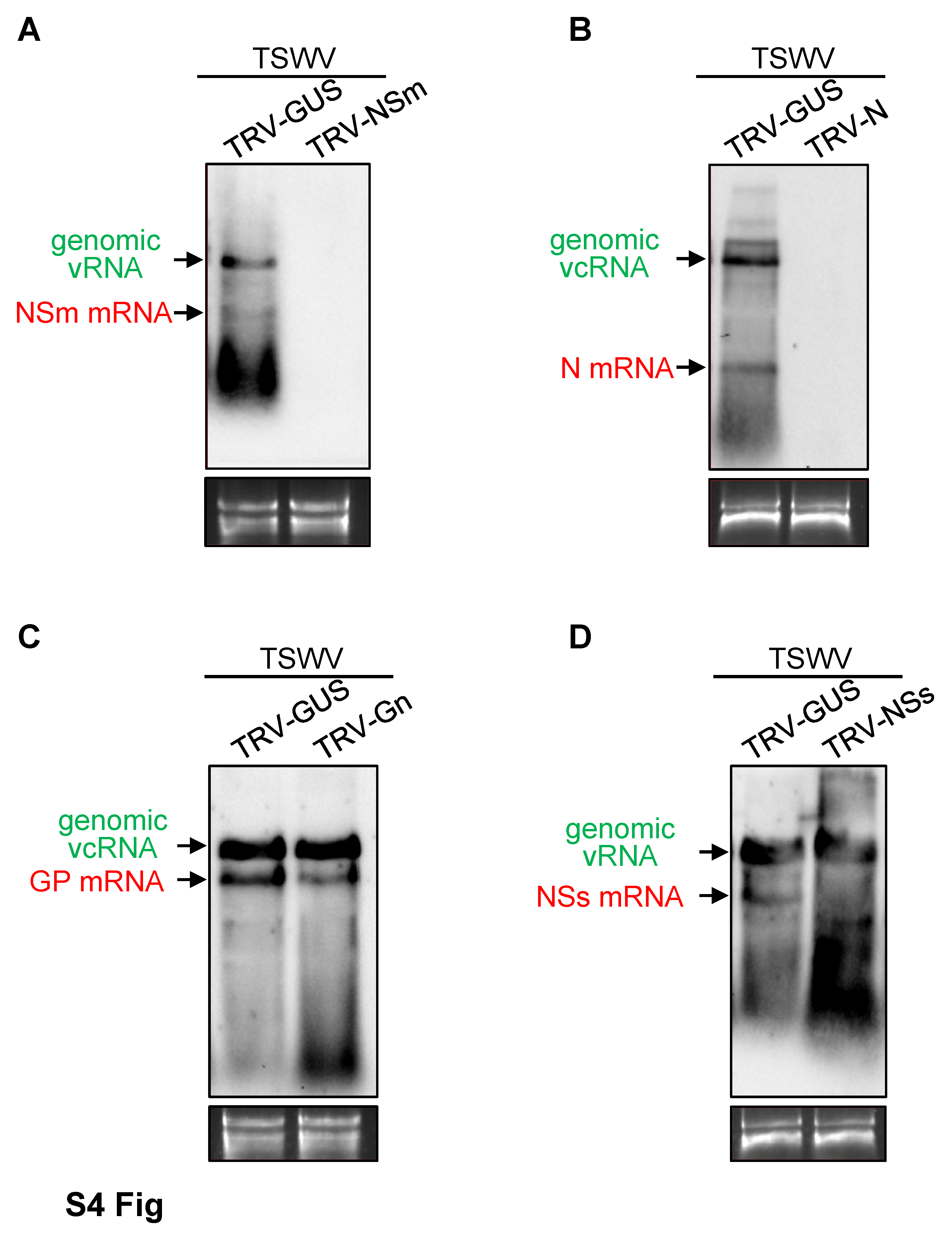

Supplement: S4 Fig — (A) Detecting genomic vRNA and NSs mRNA of TSWV S segment targeted by RISC from TRV-NSs and TRV-GUS plants using strand specific DIG-labelled NSs probe. (B) Northern blot analysis of viral genomic vcRNA and N mRNA of TSWV S segment targeted by pre-assembled RISC from TRV-GUS and TRV-N in N. benthamiana plants using strand specific DIG-labelled N probe. (C) Detection of genomic vRNA and NSm mRNA of TSWV M segment targeted by antiviral RISC from TRV-NSm and TRV-GUS pre-infected plants by northern blot analysis using strand specific DIG-labelled NSm probe. (D) Genome M vcRNA and GP mRNA were detected in TRV-Gn and TRV-GUS pre-infected plants using strand specific DIG-labelled Gn probe. N. benthamiana plants were agroinoculated with the TRV constructs and after about 15 days, to allow for pre-assembly of antiviral RISC, the local leaves were challenged with TSWV. Samples were collected at 5 dpi from the TSWV inoculated leaves of TRV pre-infected plants. Genomic vcRNA (or vRNA) and viral mRNA bands are indicated by the arrows in green and red, respectively. Ethidium bromide staining was used to show equal RNA loading of samples. (TIF) [file ppat.1009757.s004.tif]

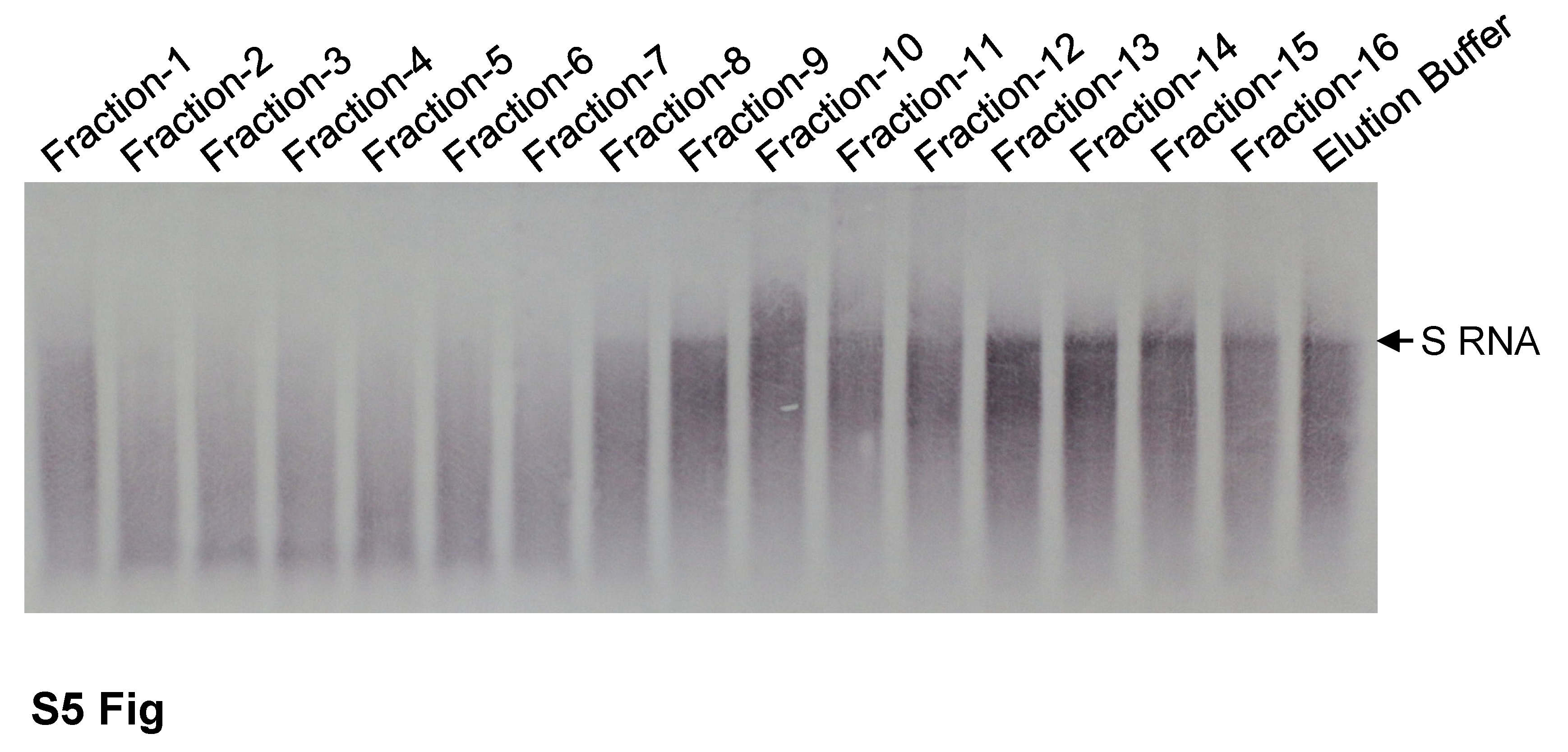

Supplement: S5 Fig — Sixteen Superdex S-200 fractions were isolated from TZSV infected N. benthamiana. Every fraction (600 μL each) was incubated in vitro with digoxigenin (DIG) labeled full length S RNA of TZSV (100 ng) to examine which fraction contained the RISC associated nuclease activity to viral S RNA. (TIF) [file ppat.1009757.s005.tif]

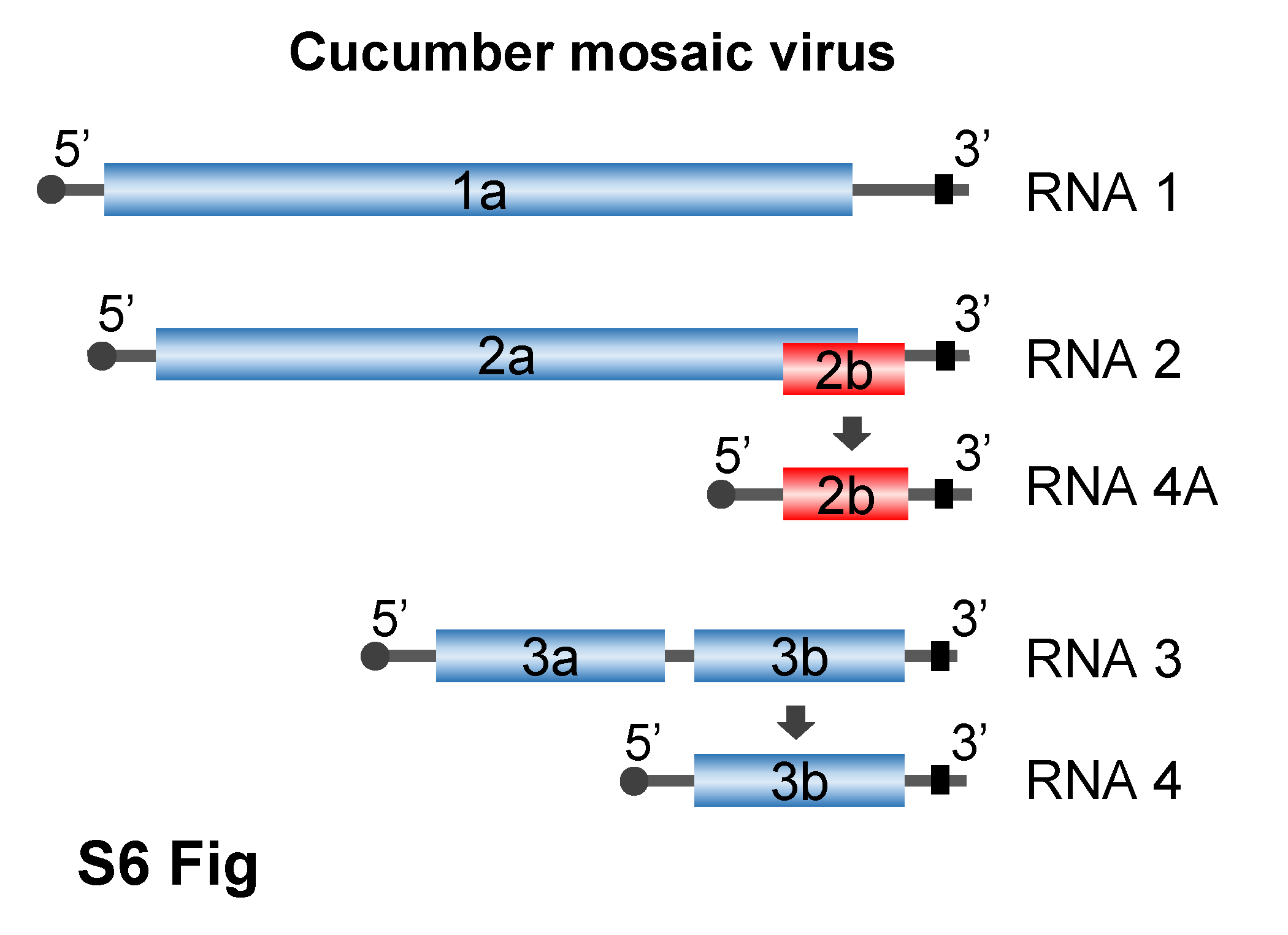

Supplement: S6 Fig — CMV RNAs encodes five open reading frames 2a is encoded by genomic RNA2 of CMV. 2b is encoded by subgenomic RNA2 of CMV. The 1a, 2a and 3a ORFs expressed from the genomic strands are denoted in blue, whereas 2b and CP, expressed from a subgenomic mRNA, are denoted in red. (TIF) [file ppat.1009757.s006.tif]
